# Systemic Inflammation Is Associated With Longitudinal Changes in Cognitive Performance Among Urban Adults

**DOI:** 10.3389/fnagi.2018.00313

**Published:** 2018-10-09

**Authors:** May A. Beydoun, Gregory A. Dore, Jose-Atilio Canas, Hailun Liang, Hind A. Beydoun, Michele K. Evans, Alan B. Zonderman

**Affiliations:** ^1^Laboratory of Epidemiology and Population Sciences, National Institute on Aging (NIA), National Institutes of Health Intramural Research Program, Baltimore, MD, United States; ^2^Johns Hopkins All Children's Hospital, St. Petersburg, FL, United States; ^3^Institute on Social Welfare, Renmin University of China, Beijing, China; ^4^Department of Medicine, Johns Hopkins School of Medicine, Baltimore, MD, United States

**Keywords:** inflammation, serum biomarkers, C-reactive protein (CRP), cognitive performance, urban adults

## Abstract

**Objectives/Background:** Systemic inflammation can affect cognitive performance over time. The current study examined associations between systemic inflammation and cognitive performance among African Americans and Whites urban adults, stratifying by sex, and age group and by race.

**Patients/Methods:** Among 1,555–1,719 White and African-American urban adults [Age_base_: 30–64y, 2004-2013, mean±SD follow-up time(y): 4.64 ± 0.93y], conducted linear mixed-effects regression models were conducted to test associations of inflammatory markers [C-reactive protein, Erythrocyte Sedimentation Rate (ESR), albumin, iron, and an inflammation composite score (ICS)] with longitudinal cognitive performance.

**Results:** Among key findings, CRP was linked to poorer baseline mental status among younger women (≤50y, γ_01_ = –0.03 ± 0.01*, p* = 0.002) and poorer attention in older women (>50y, γ_01_ = −0.024 ± 0.007*, p* < 0.004) and African-Americans (γ_01_ = −0.029 ± 0.008*, p* < 0.001). ESR was related to faster decline on verbal memory among older men (>50y, γ_11_ = −0.008 ± 0.003, *P* = 0.009); with poorer performance on attention tests overall (γ_01_ = −0.010 ± 0.003*, P* = 0.003) and among African-Americans (γ_01_ = −0.013 ± 0.004*, P* = 0.002); on verbal fluency among older women (>50y,γ_01_ = −0.037 ± 0.013*, P* = 0.004) and on executive function: overall (γ_01_ = +0.62 ± 0.21*, P* = 0.004), older men (>50y, γ_01_ = +1.69 ± 0.53*, P* = 0.001) and African-Americans (γ_01_ = +0.84 ± 0.28, *P* = 0.002). Albumin was linked to slower attention decline among older men (>50y, γ_11_ = +0.329 ± 0.103, *P* = 0.009), over-time improvement in executive function overall (γ_11_ = −6.00 ± 2.26*, P* = 0.008), and better baseline psychomotor speed among African-Americans (γ_01_ = +0.56 ± 0.19*, P* = 0.003). Finally, ICS predicted faster decline on visual memory/visuo-constructive abilities among older men (>50y, γ_11_ = +0.17 ± 0.06*, p* = 0.003).

**Conclusion:** In sum, strong associations between systemic inflammation and longitudinal cognitive performance were detected, largely among older individuals (>50y) and African-Americans. Randomized trials targeting inflammation are warranted.

## Introduction

Chronic systemic inflammation is a risk for neurodegeneration manifesting as Alzheimer's Disease (AD) and age-related cognitive decline. Markers of inflammation are associated with poorer cross-sectional cognitive performance, faster longitudinal decline in various domains of cognition (Lai et al., 2017; Stacey et al., 2017) as well as with structural and functional brain changes representing early markers of AD, including brain region activity, regional cortical thickness and white matter microstructural integrity (Jefferson et al., 2007; Hoshi et al., 2010; Wersching et al., 2010; Wada et al., 2011; Bettcher et al., 2012; Satizabal et al., 2012; Arfanakis et al., 2013; Taki et al., 2013; Walker et al., 2017; Corlier et al., 2018; Gu et al., 2018; Warren et al., 2018). However, few studies have examined cross-sectional or longitudinal associations of inflammation with cognitive performance in a bi-racial adult cohort (Yaffe et al., 2003; Windham et al., 2014; Goldstein et al., 2015; Walker et al., 2017), and none have tested effect modification by race, age, and sex in the relationship between systemic inflammation and rate of change in cognitive performance over time while using a large battery of cognitive tests.

Acute-phase markers such as C-reactive protein (CRP) increase over 1,000-folds during inflammation induced by infection, trauma, surgery, burns, tissue infarction, various immunologically mediated, and advanced cancer (Gabay and Kushner, 1999). Others including complement system proteins and ceruloplasmin increase only by ~50% (Gabay and Kushner, 1999). These changes in acute phase marker concentrations are largely due to their modified liver production (Gabay and Kushner, 1999). At higher does, CRP was shown to increase the paracellular permeability at the blood brain barrier, in the context of leptin resistance (Hsuchou et al., 2012). In contrast, cytokines are intercellular signaling polypeptides produced by activated cells, including macrophages and monocytes (Gabay and Kushner, 1999). Each cytokine has multiple sources, functions and targets, and operates both within a cascade and a network (Gabay and Kushner, 1999). Among cytokines, interleukin-6 (IL-6) is the chief stimulator of acute-phase protein production, including CRP (Gabay and Kushner, 1999). While CRP, IL-6 and other key cytokines are the focus of most recent epidemiological investigations (Bettcher et al., 2012; Satizabal et al., 2012; Trollor et al., 2012; Arfanakis et al., 2013; O'bryant et al., 2013; Taki et al., 2013; Yarchoan et al., 2013; Krogh et al., 2014; Lima et al., 2014; Metti et al., 2014; Windham et al., 2014; Goldstein et al., 2015; Matsushima et al., 2015; Palta et al., 2015; Gong et al., 2016; Tampubolon, 2016; Tegeler et al., 2016; Watanabe et al., 2016; Hsu et al., 2017; Lai et al., 2017; Walker et al., 2017; Corlier et al., 2018; Hajjar et al., 2018; Warren et al., 2018), systemic inflammation is accompanied by other changes including an increased level of fibrinogen (Gabay and Kushner, 1999; Van Oijen et al., 2005; Luciano et al., 2009; Marioni et al., 2009; Wada et al., 2011; Tampubolon, 2016), a rise in white blood cell (WBC) counts (Warren et al., 2018) and a reduction in albumin (or microalbuminuria) (Gabay and Kushner, 1999; Dik et al., 2005; Kuo et al., 2007; Vupputuri et al., 2008; Ng et al., 2009; Llewellyn et al., 2010; Onem et al., 2010; Taniguchi et al., 2014; Koyama et al., 2016; Murayama et al., 2017; Walker et al., 2017; Warren et al., 2018), and transferrin (or iron status measures) concentrations (Onem et al., 2010; Taniguchi et al., 2014; Murayama et al., 2017). Thus, a composite score summarizing inter-correlated changes is warranted yielding a clearer picture of the association between systemic inflammation and cognitive outcomes.

The current study examined associations between systemic inflammation and cognitive performance among African Americans and Whites urban adults participating in the Health Aging in Neighborhoods of Diversity across the Life Span (HANDLS) study. Markers known to either increase or decrease during inflammation (Gabay and Kushner, 1999; Walker et al., 2017) were tested against cross-sectional and longitudinal cognitive function, stratifying by key socio-demographic factors, including age, sex, and race.

## Materials and methods

### Database and study participants

Initiated in 2004, HANDLS is a prospective cohort study focusing on the cardiovascular and cognitive health of an ethnically and socio-economically diverse urban population. The study used area probability sampling to recruit a socioeconomically diverse group of African American and White urban adults (baseline age: 30–64y) who resided in thirteen Baltimore city, MD neighborhoods (Evans et al., 2010). The present study included data from the baseline visit 1 (2004-2009) and the first follow-up examination (visit 2; 2009-2013), with follow-up time ranging between <1 and ~8y, mean±SD of 4.64 ± 0.93y. Data included a battery of cognitive tests measured at both visits and markers of inflammation measured at the baseline visit 1, as well as numerous baseline or fixed covariates. The study obtained written informed consent from all participants who were additionally provided with a protocol booklet and a video explaining key study procedures. The National Institute on Environmental Health Sciences Institutional Review Board of the National Institutes of Health approved the study protocol. Moreover, the HANDLS staff and investigators are required to adhere to NIH's biosecurity and safety procedures. They receive mandatory annual refresher training. They are also inspected by NIH's safety officers and by medical records compliance officers. In addition, HANDLS staff and investigators use universal precautions in handling all biomaterials.

The initial sample of HANDLS included 3,720 participants (Phase I, visit 1). At Phase II of visit 1, participant examinations yielded data on biochemical indices and cognitive performance for a sub-set of the initial sample. Specifically, baseline CRP, erythrocyte sedimentation rate (ESR), albumin, and serum iron were available on 2,646, 2,709, 2,753, and 2,749 participants, respectively. Consequently, the main exposure [a composite of all 4 measures, inflammation composite score (ICS)] was available among 2,580 participants at baseline. Sample sizes varied for the cognitive tests. Consequently, we determined the size of the final analytic sample based on exposure and covariate non-missingness at baseline and cognitive performance measure non-missingness at either visit. Figure S1 describes sample selection for all exposures as well as the main composite exposure. The final analytic sample sizes ranged between 1,555 and 1,719 participants with *k* = 1.5–1.7 observation/participant.

### Cognitive assessment

The present study assessed cognitive performance using 7 tests that yielded 11 test scores, tapping into 7 distinctive domains (Global, attention, learning/memory, executive function, visuo-spatial/visuo-construction ability, psychomotor speed, language/verbal): the Mini-Mental State Examination (MMSE), the California Verbal Learning Test (CVLT) immediate (List A) and Delayed Free Recall (DFR), Digit Span Forward and Backwards tests (DS-F and DS-B), the Benton Visual Retention Test (BVRT), Animal Fluency test (AF), Brief Test of Attention (BTA), Trails A and B and the Clock Drawing Test (CDT) (Supplemental Method 1). All participants were able to complete informed consent after being probed for understanding the protocol. Despite the lack of dementia diagnosis, all participants were screened using the MMSE as a global mental status test, which they completed successfully (total score ≥24). In cases where MMSE was low (~6.6% were <24 at visit 1 and 1.9% at visit 2), it was judged to be caused by poor literacy rather than being a sign of dementia.

### Measures of inflammation and composite score

All laboratory tests selected for this study were done at Quest Diagnostics, Chantilly, VA. Using 5 mL of refrigerated whole blood stored in lavender-top EDTA tube, the Erythrocyte Sedimentation Rate (ESR) was tested within 24 h of blood draw. The blood draw was done in the early morning in a fasting state before the participant was offered breakfast. This test used automated modified Westergren photochemical capillary stopped flow kinetic analysis. The Mayo clinic reports a reference of 0–22 mm/h for men and 0–29 mm/h for women. (https://www.mayoclinic.org/tests-procedures/sed-rate/about/pac-20384797) and is considered a proxy measure for serum fibrinogen (Yin et al., 2017). Similarly, high sensitivity CRP (hs-CRP) was analyzed with an immunoturbidimeter (Siemens/Behring Nephelometer II), using 0.5–1 mL of plasma, with the range 1–10 mg/dL indicating average or high cardiovascular risk and >10 mg/dL suggestive of an infection or a chronic inflammation. Using 0.5–1 mL sample of plasma prepared with heparin and refrigerated for up to 30 days, albumin was measured with spectrophotometry, with an expected reference range of 3.6–5.1 g/dL. Finally, for serum iron, 0.5–1 mL of fasting serum was collected, transported at room temperature (with heparin added) and refrigerated or frozen subsequently. Serum iron was also measured with spectrophotometry, with reference ranges for men aged ≥30y set at 50–180 mcg/dL and for women: 20–49y (40–190 mcg/dL) and 50+y(45–160 mcg/dL). All markers were used as continuous untransformed variables in the main analysis. A summary score of inflammation, namely the z- inflammation composite score (ICS) combined all 4 individual measures using a principal components analysis extracting one component score (a z-score) that explained >40% of the total variance. The ICS was used in the main analysis.

### Covariates

Covariates included in our main models were selected based on their well-known association with the outcome of interest, namely cognitive decline (Barnes and Yaffe, 2011). Among those covariates, socio-demographic characteristics included baseline age, sex, race (White vs. African American), marital status, educational attainment (< High School (HS); HS, >HS) and poverty income ratio (PIR <125% for “poor”). Age group was categorized as >50 vs. ≤ 50y, when used as an effect modifier combined with sex, but was entered as a continuous variable in models. Lifestyle and health-related covariates included measured body mass index (BMI, kg/m^2^), self-reported opiate, marijuana, or cocaine use (“current” vs. “never or former”), smoking status (“current” vs. “never or former”), and the Wide Range Achievement Test (WRAT) letter and word reading subtotal scores to measure literacy. (See Supplemental Method 1) Depressive symptoms, mainly affective depressed mood were measured using the 20-item Center for Epidemiological Studies-Depression scale (CES-D). Baseline CES-D total score was included in the analysis as a potential confounder in the association between inflammation and cognitive change or baseline performance. Overall dietary quality was assessed using the total score from the Healthy Eating Index (HEI-2010), based on two self-reported 24-h recalls administered at baseline. Steps for calculating HEI-2010 are outlined in: http://appliedresearch.cancer.gov/tools/hei/tools.html and http://handls.nih.gov/06Coll-dataDoc.html. Finally, first-visit self-reported history of type 2 diabetes, hypertension, dyslipidemia, cardiovascular disease (stroke, congestive heart failure, non-fatal myocardial infarction. or atrial fibrillation), inflammatory disease (multiple sclerosis, systemic lupus, gout, rheumatoid arthritis, psoriasis, Thyroid disorder. and Crohn's disease), and use of non-steroidal anti-inflammatory drugs (NSAIDs, prescription, and over-the-counter) over the past 2 weeks, were considered as covariates, as was done in previous studies (Gimeno et al., 2009; Bettcher et al., 2012).

### Statistical analysis

All analyses were carried out with Stata release 15.0 (STATA, 2017). Accounting for sampling weights, population means and proportions were estimated. While means across key stratifying variables (e.g., age/sex or race) were contrasted using svy:reg, comparisons between categorical variables were accomplished using svy:tab and design-based *F*-tests. The main analysis included a series of mixed-effects regression models with 11 continuous cognitive test score as outcomes. In each of those models, the *TIME* variable, expressed as years elapsed between data waves, was entered as a fixed and random effect (along with the intercept) and was interacted with several covariates including the main exposure variable, namely the inflammation composite score. All mixed-effects regression models assumed that the outcome was missing at random with repeated measures of ~1.5–1.7 visits/person and accounted for variable time of follow-up (See Supplemental Method 2) (Ibrahim and Molenberghs, 2009). Moreover, to visualize key findings from mixed-effects regression models, predictive margins of outcomes were estimated and plotted across *TIME* (y), stratifying by exposure levels (−1 = mean-1 SD, 0 = mean, +1 = mean+1 SD). Mixed-effects regression models were also conducted to test longitudinal associations of each of the 4 inflammation markers with the 11 continuous cognitive test scores.

Simultaneous moderating effects of sex and age was tested by adding interaction terms to separate multivariable mixed-effects regressions (3-way and 4-way interaction terms between *TIME*, exposure, Age group, and sex) and by stratifying the models by sex/age group, thus testing main associations within each of the following groups: (1) Younger men (≤ 50y), (2) Older men (>50y), (3) Younger women (≤ 50y), (4) Older women (>50y). A similar approach was adopted for stratification by race: [(1) Whites, (2) African-Americans] (Supplemental Method 2), given the previously reported differences in inflammatory markers by age, sex, and race groups (Mcconnell et al., 2002; Herd et al., 2012; Lu et al., 2017).

Non-random selection of participants may lead to selection bias due to systematic differences between the selected group and the target population on major characteristics. To account for this bias in each mixed-effect regression model, a 2-stage Heckman selection process was carried out. At a first sage, a probit model with binary outcome being selected = 1 vs. unselected = 0, was conducted to compute an inverse mills ratio (derived from the predicted probability of being selected, conditional on the covariates baseline age, sex, race, poverty status, and education). At a second stage, this inverse mills ratio was included in the final mixed-effects regression model as a covariate, similar to prior studies (Beydoun et al., 2013).

In all our analyses, we chose a type I error of 0.05 for main effects and 0.10 for interaction terms (Selvin, 2004), prior to correcting for multiple testing. A familywise Bonferroni procedure was adopted for multiple testing correction by accounting only for cognitive test multiplicity with the assumption that each exposure constitutes a distinctive substantive hypothesis.(Hochberg and Tamhane, 1987) Therefore, for main effects, *p* < 0.0045 (0.05/11) was considered significant, while 2-way interactions had a critical *p*-values reduced to (0.10/11 = 0.0090). Finally, 3-way and 4-way interaction terms had their critical *p*-value reduced to 0.05. This approach was adopted in at least two previous studies (Beydoun et al., 2015, 2016).

## Results

Baseline study sample characteristics are outlined in Table 1, both by age group and sex, and by race. Older participants (>50y, both sexes) had lower educational attainment and income compared to their younger counterparts (≤50y), a differential observed also among African-Americans vs. Whites. Other important differences were a lower literacy (WRAT total score) among African-Americans vs. Whites, a higher prevalence of current smoking and drug use among younger men (≤50y) vs. at least one other group, with a similar pattern observed among African-Americans vs. Whites. Both BMI and HEI-2010 were the lowest in younger men (≤50y). HEI-2010 suggested a better overall dietary quality among Whites compared with African-Americans. Generally, younger men (≤50y) reported the least number of chronic conditions, including diabetes, hypertension, dyslipidemia, cardiovascular disease, and inflammatory conditions. African-Americans' prevalence of hypertension and cardiovascular disease were higher than among Whites, while the reverse was true for dyslipidemia. NSAIDs were more likely used by older individuals (>50y), with no racial differences detected. Except for CRP, all markers reflected higher inflammation among African-Americans. In general, women had more systemic inflammation, particularly compared with younger men (≤50y).

**Table 1:** Selected baseline (Visit 1) and time-dependent study participant characteristics by age group/sex, and by race for HANDLS participants with complete and reliable baseline MMSE scores (*n* = 2,574)^a^.

	**All**	**Older women (>50y)**	**Older men (>50y)**	**Younger women (≤50y)**	**Younger men (≤50y), referent**	***P*_*age*×*sex*_^b^**	**Whites**	**African-Americans**	***P*_race_^c^**
*%*±SE		20.9 ± 1.2	18.3 ± 1.1	33.6 ± 1.7	27.2 ± 1.6		36.4 ± 1.5	63.6 ± 1.5	
	(*N* = 2,574)	(*N* = 668)	(*N* = 511)	(*N* = 792)	(*N* = 603)		(*N* = 1,107)	(*N* = 1,467)	
Age at baseline, y	46.9 ± 0.3	56.7 ± 0.3^d^	56.5 ± 0.3^d^	40.5 ± 0.4	40.7 ± 0.4	<0.001	46.7 ± 0.4	47.0 ± 0.4	0.52
	(*N* = 2,574)	(*N* = 668)	(*N* = 511)	(*N* = 792)	(*N* = 603)		(*N* = 1,107)	(*N* = 1,467)	
Sex, % male	45.0 ± 1.8	__	__	__	__		46.8 ± 2.1	44.7 ± 2.4	0.52
	(*N* = 2,574)						(*N* = 1,107)	(*N* = 1,467)	
Married, %	35.1 ± 1.7	35.4 ± 3.4	38.8 ± 3.3	29.9 ± 2.9^d^	39.1 ± 3.5	0.10	45.1 ± 2.3	29.7 ± 2.2	<0.001
	(*N* = 2,397)	(*N* = 602)	(*N* = 462)	(*N* = 760)	(*N* = 572)		(*N* = 1,007)	(*N* = 1,390)	
**EDUCATION, %**									
<HS	4.2 ± 0.5	6.2 ± 1.4^d^	7.7 ± 1.6^d^	2.5 ± 0.6	2.4 ± 0.7	0.011	5.1 ± 0.8	3.7 ± 0.7	<0.001
HS	52.5 ± 1.7	45.4 ± 3.1^d^	45.4 ± 3.3^d^	55.6 ± 3.3	58.9 ± 3.4		40.2 ± 2.0	59.6 ± 2.4	
>HS	38.8 ± 1.7	43.6 ± 3.3^d^	42.8 ± 3.4^d^	38.2 ± 3.2	33.2 ± 3.2		47.0 ± 2.2	34.1 ± 2.3	
Missing	4.5 ± 0.8	4.8 ± 1.2^d^	4.1 ± 1.2^d^	3.7 ± 1.5	5.6 ± 2.0		7.7 ± 1.1	2.6 ± 1.2	
	(*N* = 2,574)	(*N* = 668)	(*N* = 511)	(*N* = 792)	(*N* = 603)		(*N* = 1,107)	(*N* = 1,467)	
Literacy (WRAT score)	43.3 ± 0.2	42.9 ± 0.4	42.2 ± 0.6	43.7 ± 0.4	43.7 ± 0.6	0.08	46.8 ± 0.3	41.2 ± 0.3	<0.001
	(*N* = 2,560)	(*N* = 664)	(*N* = 508)	(*N* = 788)	(*N* = 600)		(*N* = 1,103)	(*N* = 1,457)	
PIR < 125%, %	19.4 ± 1.0	22.4 ± 2.2^d^	16.4 ± 1.7	22.0 ± 2.1^d^	16.0 ± 1.6	0.020	12.2 ± 0.9	23.5 ± 1.5	<0.001
	(*N* = 2,574)	(*N* = 668)	(*N* = 511)	(*N* = 792)	(*N* = 603)		(*N* = 1,107)	(*N* = 1,467)	
**CURRENT SMOKING STATUS, %**									
Currently smoking	43.3 ± 1.7	31.7 ± 3.2^d^	43.1 ± 3.4	42.2 ± 3.2	53.9 ± 3.4	0.003	35.7 ± 2.0	47.8 ± 2.4	<0.001
Missing	5.0 ± 0.8	7.6 ± 2.1^d^	4.4 ± 1.4	5.0 ± 1.6	3.3 ± 1.5		3.6 ± 2.0	5.8 ± 1.3	
	(*N* = 2,574)	(*N* = 667)	(*N* = 511)	(*N* = 792)	(*N* = 603)		(*N* = 1,107)	(*N* = 1,467)	
**CURRENT USE OF ILLICIT DRUGS**, ***%***									
Used any type	48.8 ± 1.7	31.3 ± 3.2^d^	54.4 ± 3.3^d^	43.3 ± 3.3^d^	65.1 ± 3.3	<0.001	41.0 ± 2.1	53.2 ± 2.4	<0.001
Missing	7.9 ± 0.8	10.3 ± 2.2^d^	8.8 ± 1.8^d^	8.0 ± 1.6^d^	5.3 ± 1.1		11.1 ± 1.3	6.1 ± 1.1	
	(*N* = 2,574)	(*N* = 668)	(*N* = 511)	(*N* = 792)	(*N* = 603)		(*N* = 1,107)	(*N* = 1,467)	
Body mass index, *kg.m^−2^*	29.7 ± 0.3	31.7 ± 0.6^d^	28.9 ±± 0.4^d^	30.7 ± 0.6^d^	27.5 ± 0.5	<0.001	29.2 ± 0.3	30.0 0.4	0.14
	(*N* = 2,574)	(*N* = 668)	(*N* = 511)	(*N* = 792)	(*N* = 603)		(*N* = 1,107)	(*N* = 1,467)	
**HEI-2010 total score**	43.8 ± 0.4	47.6 ± 0.9^d^	44.3 ± 0.8^d^	42.6 ± 0.7 ^d^	42.2 ± 0.7	<0.001	45.2 ± 0.6	43.0 ± 0.5	0.006
	(*N* = 1,996)	(*N* = 506)	(*N* = 382)	(*N* = 640)	(*N* = 468)		(*N* = 856)	(*N* = 1,140)	
**DEPRESSIVE SYMPTOMS**									
CES-D score	13.8 ± 0.4	15.1 ± 0.7^d^	12.4 ± 0.6	14.8 ± 0.8^d^	12.4 ± 0.6	0.07	13.4 ± 0.4	14.0 ± 0.5	0.44
	(*N* = 2,558)	(*N* = 663)	(*N* = 508)	(*N* = 787)	(*N* = 600)		(*N* = 1,100)	(*N* = 1,458)	
Diabetes, %	12.7 ± 1.1	23.5 ± 2.9^d^	19.8 ± 2.8^d^	7.0 ± 1.4	7.0 ± 1.8	<0.001	10.6 ± 1.3	13.9 ± 1.5	0.10
	(*N* = 2,404)	(*N* = 626)	(*N* = 482)	(*N* = 737)	(*N* = 559)		(*N* = 1,032)	(*N* = 1,372)	
Hypertension, %	36.9 ± 1.7	57.0 ± 3.4^d^	53.6 ± 3.6^d^	30.1 ± 3.4^d^	18.8 ± 2.7	<0.001	27.3 ± 1.9	42.1 ± 2.5	<0.001
	(*N* = 2,281)	(*N* = 605)	(*N* = 461)	(*N* = 693)	(*N* = 522)		(*N* = 981)	(*N* = 1,300)	
Dyslipidemia, %	23.5 ± 1.4	37.0 ± 3.0^d^	35.3 ± 3.3^d^	14.2 ± 2.3	16.7 ± 2.9	<0.001	27.8 ± 2.0	21.2 ± 1.9	0.018
	(*N* = 2,282)	(*N* = 602)	(*N* = 463)	(*N* = 694)	(*N* = 523)		(*N* = 982)	(*N* = 1,300)	
Cardiovascular disease^e^, %	10.9 ± 1.0	20.1 ± 2.6^d^	16.6 ± 2.7^d^	7.9 ± 1.5	4.0 ± 1.4	<0.001	8.0 ± 1.1	12.4 ± 1.4	0.010
	(*N* = 2,410)	(*N* = 626)	(*N* = 483)	(*N* = 738)	(*N* = 563)		(*N* = 1,035)	(*N* = 1,375)	
Inflammatory conditions^f^, %	13.1 ± 1.1	23.0 ± 2.5^d^	14.3 ± 2.5^d^	12.3 ± 2.3^d^	5.9 ± 1.4	<0.001	14.5 ± 1.5	12.3 ± 1.5	0.30
	(*N* = 2,404)	(*N* = 626)	(*N* = 482)	(*N* = 736)	(*N* = 560)		(*N* = 1,032)	(*N* = 1,372)	
NSAIDS^g^, %	20.7 ± 1.5	26.4 ± 2.7^d^	32.0 ± 3.4^d^	18.3 ± 3.0	12.0 ± 2.5	<0.001	20.8 ± 1.9	20.7 ± 2.0	0.97
	(*N* = 2,417)	(*N* = 629)	(*N* = 486)	(*N* = 738)	(*N* = 564)		(*N* = 1,041)	(*N* = 1,376)	
C-reactive protein, mg/dL	4.39 ± 0.32	5.86 ± 0.54^d^	4.26 ± 1.32	4.57 ± 0.42^d^	3.09 ± 0.39	0.001	3.8 ± 0.2	4.7 ± 0.5	0.080
	(*N* = 2,349)	(*N* = 618)	(*N* = 460)	(*N* = 721)	(*N* = 550)		(*N* = 1,022)	(*N* = 1,327)	
Erythrocyte Sedimentation Rate, ESR	16.7 ± 0.7	22.4 ± 1.2^d^	13.3 ± 1.2	19.6 ± 1.1^d^	10.8 ± 1.4	<0.001	10.9 ± 0.4	20.1 ± 1.0	<0.001
	(*N* = 2,414)	(*N* = 636)	(*N* = 480)	(*N* = 733)	(*N* = 565)		(*N* = 1,051)	(*N* = 1,363)	
Serum albumin	4.27 ± 0.01	4.27 ± 0.02^d^	4.28 ± 0.02^d^	4.18 ± 0.02^d^	4.39 ± 0.03	0.016	4.37 ± 0.01	4.21 ± 0.02	<0.001
	(*N* = 2,452)	(*N* = 644)	(*N* = 483)	(*N* = 752)	(*N* = 573)		(*N* = 1,079)	(*N* = 1,373)	
Serum iron	83.0 ± 1.2	80.5 ± 1.8^d^	93.5 ± 2.6	72.7 ± 2.1^d^	90.4 ± 2.8	0.37	90.5 ± 1.7	78.3 ± 1.6	<0.001
	(*N* = 2,447)	(*N* = 641)	(*N* = 483)	(*N* = 750)	(*N* = 573)		(*N* = 1,078)	(*N* = 1,369)	
Inflammation composite score, *z*-score	−0.014 ± 0.050	+0.284 ± 0.093^d^	−0.288 ± 0.109	+0.353 ± 0.074^d^	−0.516 ± 0.113	<0.001	−0.518 ± 0.050	+0.270 ± 0.069	<0.001
	(*N* = 2,293)	(*N* = 603)	(*N* = 452)	(*N* = 698)	(*N* = 540)		(*N* = 994)	(*N* = 1,299)	

*CES-D, Center for Epidemiologic Studies-Depression; MMSE, Mini-Mental State Examination; PIR, poverty income ratio; WRAT, Wide Range Achievement Test*.

a *Values are weighted mean ± SEM or percent±SEP. Largest sample size is N = 2,574*.

b *P-value was based on linear regression models when row variable is continuous (svy:reg) with sex/age group coded as continuous variable (0 = younger men, 1 = younger women, 2 = older men, 3 = older women) and design-based F-test when row variable is categorical (svy:tab)*.

c *P-value was based on linear regression models when row variable is continuous (svy:reg) with race group coded as continuous variable (0 = Whites, 1 = African-Americans) and design-based F-test when row variable is categorical (svy:tab)*.

d *P < 0.05. P-value was based on linear regression models when row variable is continuous (svy:reg) and design-based F-test when row variable is categorical (svy:tab), comparing each of the sex/age categories to the referent category of younger men (≤50y)*.

e *Cardiovascular disease include self-reported stroke, congestive heart failure, non-fatal myocardial infarction, or atrial fibrillation*.

f *Inflammatory conditions include multiple sclerosis, systemic lupus, gout, rheumatoid arthritis, psoraiasis, Thyroid disorder and Crohn's disease*.

g *Non-steroidal anti-inflammatory drugs (NSAIDS) include over the counter and prescription drugs in that category*.

Table S1 shows marked racial disparities in cognitive performance, which persisted over the two waves of data and with poorer performance observed among African-Americans. Of the 11 tests, however, only three indicated a marked decline in cognitive performance over time, while one (MMSE total score) suggested a learning effect among Whites only.

A series of mixed-effects linear regression models (Table 2, Tables S2–S5) were conducted to test our main hypotheses. After correction for multiple testing, a higher baseline ICS was associated with a faster decline on a test of visual memory/visuo-constructive abilities (BVRT), among older men only (>50y, γ_11_ = +0.17 ± 0.06*, p* = 0.003).

**Table 2:** Cognitive performance test scores by inflammation composite score (ICS), stratified by age group/sex and by race, for HANDLS participants with complete and reliable baseline and/or follow-up cognitive scores: mixed-effects regression models^a^.

	**All**	**Older women(>50y)**	**Older men (>50y)**	**Younger women (≤50y)**	**Younger men (≤50y)**	**Whites**	**African- Americans**
**MINI-MENTAL STATE EXAM, TOTAL SCORE**							
Intercept	+**26.6 ± 0.2**^b^	+**27.9 ± 0.4**^b^	+**27.9 ± 0.4** ^b^	+**27.1 ± 0.3**^b^	+**25.7 ± 0.6**^b^	+**27.0 ± 0.2**^b^	+**26.1 ± 0.3**^b^
TIME	+**0.11 ± 0.06** ^b^	+0.03 ± 0.12	+0.17 ± 0.15	+0.08 ± 0.09	+0.20 ± 0.14	+0.09 ± 0.07	+0.12 ± 0.08
ICS	−0.045 ± 0.036	+0.049 ± 0.067	***+0.17 ± 0.09***	−0.11 ± 0.06	+0.09 ± 0.07	+0.04 ± 0.06	+0.04 ± 0.05
ICS × TIME	−0.012 ± 0.010	−0.017 ± 0.020 ^e^	−0.028 ± 0.028	−0.004 ± 0.018	+0.004 ± 0.018	−0.015 ± 0.016	+0.009 ± 0.013
	(*N* = 1,659; *k* = 1.7)	(*N* = 429, *k* = 1.7)	(*N* = 332, *k* = 1.6)	(*N* = 510, *k* = 1.7)	(*N* = 388, *k* = 1.6)	(*N* = 724, *k* = 1.6)	(*N* = 935 *k* = 1.7)
**CALIFORNIA VERBAL LEARNING TEST (CVLT), LIST A**							
Intercept	+**24.6 ± 0.7**^b^	+**24.8 ± 1.5**^b^	+**20.8 ± 1.4**^b^	+**23.6 ± 3.2**^b^	+**21.6 ± 2.0**^b^	+**25.4 ± 1.0**^b^	+**22.1 ± 1.0**^b^
TIME	−**1.42 ± 0.17**^b^	−**1.31 ± 0.38**^b^	−**1.83 ± 0.35**^b^	−**1.80 ± 0.28**^b^	−0.98 ± 0.59	−**1.65 ± 0.27**^b^	−**1.06 ± 0.22**^b^
ICS	−0.040 ± 0.133	−0.14 ± 0.25	+0.12 ± 0.24	−0.45 ± 0.29^d^	+0.32 ± 0.27	−0.02 ± 0.24	+0.04 ± 0.16
ICS × TIME	+0.007 ± 0.033	+0.071 ± 0.068	−0.021 ± 0.072	+0.023 ± 0.064	−0.031 ± 0.062	+0.091 ± 0.059	−0.032 ± 0.039
	(*N* = 1,588, *k* = 1.6)	(*N* = 411, *k* = 1.6)	(*N* = 315, *k* = 1.5)	(*N* = 496, *k* = 1.6)	(*N* = 366, *k* = 1.6)	(*N* = 689, *k* = 1.5)	(*N* = 899, *k* = 1.6)
**CVLT, FREE DELAYED RECALL**							
Intercept	+**7.8 ± 0.3**^b^	+**7.2 ± 0.8**^b^	+**7.2 ± 0.8**^b^	+**8.0 ± 0.7**^b^	+**6.7 ± 1.0**^b^	+**7.8 ± 0.5**^b^	+**6.9 ± 0.5**^b^
TIME	−**0.49 ± 0.08**^b^	−**0.59 ± 0.18**^b^	−**0.47 ± 0.16**^b^	−**0.50 ± 0.13**^b^	−**0.67 ± 0.28** ^b^	−**0.45 ± 0.13** ^b^	−**0.46 ± 0.11**^b^
ICS	−0.01 ± 0.02	−0.12 ± 0.12	+0.13 ± 0.11	−**0.37 ± 0.13**^b, d^	−0.17 ± 0.14	−0.05 ± 0.11	−**0.16 ± 0.08**^b^
ICS × TIME	+0.008 ± 0.016	+0.043 ± 0.029	−0.044 ± 0.034	+0.003 ± 0.032	+0.03 ± 0.03	+0.017 ± 0.029	−0.008 ± 0.020
	(*N* = 1,555, *k* = 1.5)	(*N* = 405, *k* = 1.6)	(*N* = 302, *k* = 1.5)	(*N* = 490, *k* = 1.6)	(*N* = 358, *k* = 1.5)	(*N* = 669, *k* = 1.5)	(*N* = 886, *k* = 1.6)
**BENTON VISUAL RETENTION TEST**							
Intercept	+**9.3 ± 0.5**^b^	+**9.6 ± 1.1**^b^	+**9.2 ± 1.2**^b^	+**8.3 ± 1.0**^b^	+**6.5 ± 1.3**^b^	+**8.1 ± 0.7**^b^	+**10.3 ± 0.8**^b^
TIME	+**0.37 ± 0.13**^b^	+0.14 ± 0.20	+0.51 ± 029	+**0.44 ± 0.21**^b^	+**0.89 ± 0.36**^b^	+0.34 ± 0.18	+**0.66 ± 0.19**^b^
ICS	−0.05 ± 0.10	−0.12 ± 0.20	−0.18 ± 0.20	+0.23 ± 0.19^d^	−0.30 ± 0.18	−0.09 ± 0.15	−0.07 ± 0.13
ICS × TIME	+**0.049 ± 0.024**^b^	−0.002 ± 0.057	**+0.17 ± 0.06**^b^^,^ ^c^	+0.057 ± 0.042	+0.022 ± 0.039	+0.020 ± 0.037	+0.072 ± 0.033
	(*N* = 1,663, *k* = 1.7)	(*N* = 428, *k* = 1.7)	(*N* = 333, *k* = 1.6)	(*N* = 512, *k* = 1.7)	(*N* = 390, *k* = 1.7)	(*N* = 727, *k* = 1.7)	(*N* = 936, *k* = 1.7)
**BRIEF TEST OF ATTENTION**							
Intercept	+**6.6 ± 0.3**^b^	+**6.9 ± 0.5**^b^	+**6.4 ± 0.5**^b^	+**6.7 ± 0.5**^b^	+**6.7 ± 0.5**^b^	+**6.8 ± 0.3**^b^	+**5.8 ± 0.4**^b^
TIME	−0.11 ± 0.06	−0.19 ± 0.13	−0.09 ± 0.15	−0.03 ± 0.11	−0.01 ± 0.19	−0.13 ± 0.09	−0.06 ± 0.09
ICS	−0.08 ± 0.05	−0.17 ± 0.09	+0.00 ± 0.10	−0.08 ± 0.09	−0.02 ± 0.09	−0.05 ± 0.07	−0.10 ± 0.06
ICS × TIME	+0.006 ± 0.012	+0.010 ± 0.023	−0.035 ± 0.031	+0.016 ± 0.022	+0.018 ± 0.023	−0.009 ± 0.021	−0.015 ± 0.015
	(*N* = 1,604, *k* = 1.6)	(*N* = 411, *k* = 1.6)	(*N* = 322, *k* = 1.5)	(*N* = 494, *k* = 1.6)	(*N* = 377, *k* = 1.6)	(*N* = 695, *k* = 1.6)	(*N* = 909, *k* = 1.6)
**ANIMAL FLUENCY**							
Intercept	+**17.6 ± 0.6**^b^	+**17.5 ± 1.1**^b^	+**15.6 ± 1.2**^b^	+**18.6 ± 1.1**^b^	+**18.9 ± 1.7**^b^	**17.4 ± 0.8**^b^	+**16.5 ± 0.8**^b^
TIME	−0.08 ± 0.12	+0.45 ± 0.25	+0.26 ± 0.28	−0.09 ± 0.22	−**0.83 ± 0.39**^b^	+0.14 ± 0.20	−0.24 ± 0.16
ICS	−**0.21 ± 0.10**^b^	−0.23 ± 0.19	+0.25 ± 0.21	−**0.42 ± 0.21**^b^	−0.29 ± 0.23	+0.03 ± 0.19	+0.014 ± 0.027
ICS × TIME	+0.002 ± 0.023	+0.070 ± 0.043	−0.058 ± 0.056	+0.019 ± 0.043	−0.026 ± 0.049	+0.032 ± 0.044	+0.014 ± 0.027
	(*N* = 1,670, *k* = 1.7)	(*N* = 430, *k* = 1.7)	(*N* = 339, *k* = 1.7)	(*N* = 512, *k* = 1.7)	(*N* = 389, *k* = 1.7)	(*N* = 728, *k* = 1.7)	(*N* = 942, *k* = 1.7)
**DIGITS SPAN, FORWARD**							
Intercept	+**6.8 ± 0.2**^b^	+**6.6 ± 0.4**^b^	+**6.6 ± 0.5**^b^	+**6.7 ± 0.5**^b^	+**7.5 ± 0.7**^b^	+**6.9 ± 0.3**^b^	+**6.6 ± 0.3**^b^
TIME	+0.00 ± 0.05	+0.02 ± 0.08	+0.05 ± 0.12	−0.14 ± 0.10	−0.00 ± 0.16	+0.00 ± 0.09	−0.03 ± 0.06
ICS	−0.08 ± 0.04	−0.01 ± 0.02	−0.16 ± 0.08	+0.03 ± 0.09	−**0.18 ± 0.09**^b^	−0.04 ± 0.07	−0.09 ± 0.05
ICS × TIME	−0.006 ± 0.010	+0.005 ± 0.018	+0.009 ± 0.023	−0.017 ± 0.020	−0.000 ± 0.017	−0.024 ± 0.020	+0.002 ± 0.011
	(*N* = 1,664, *k* = 1.6)	(*N* = 427, *k* = 1.7)	(*N* = 334, *k* = 1.6)	(*N* = 513, *k* = 1.7)	(*N* = 390, *k* = 1.6)	(*N* = 721, *k* = 1.6)	(*N* = 943, *k* = 1.7)
**DIGITS SPAN, BACKWARD**							
Intercept	+1.22 ± 4.96	+25.5 ± 15.5	+9.2 ± 18.7	+8.66 ± 10.88	−7.42 ± 14.43	+0.41 ± 7.86	+2.51 ± 6.67
TIME	−0.11 ± 1.22	−4.21 ± 0.06	−2.50 ± 4.88	+0.54 ± 2.46	+3.73 ± 3.11	−0.95 ± 1.96	−0.24 ± 1.68
ICS	−0.03 ± 0.04	−0.13 ± 0.07	−0.09 ± 0.08	−0.04 ± 0.08	−0.08 ± 0.09	+0.07 ± 0.07	−0.07 ± 0.05
ICS × TIME	+0.008 ± 0.010	−0.010 ± 0.021	−0.019 ± 0.023	−0.006 ± 0.018	−0.006 ± 0.017	−0.020 ± 0.018	−0.005 ± 0.011
	(*N* = 1,666, *k* = 1.6)	(*N* = 427, *k* = 1.7)	(*N* = 334, *k* = 1.6)	(*N* = 514, *k* = 1.7)	(*N* = 391, *k* = 1.6)	(*N* = 723, *k* = 1.6)	(*N* = 943, *k* = 1.7)
**CLOCK, COMMAND**							
Intercept	+**8.74 ± 0.14**^b^	+**8.78 ± 0.29**^b^	+**8.63 ± 0.28**^b^	+**9.02 ± 0.25**^b^	+**8.67 ± 0.39**^b^	+**9.03 ± 0.19**^b^	+**8.24 ± 0.20**^b^
TIME	−0.05 ± 0.04	−0.12 ± 0.08	+0.02 ± 0.09	−**0.08 ± 0.05**^b^	+0.02 ± 0.05	−0.05 ± 0.06	−0.04 ± 0.05
ICS	−0.01 ± 0.03	+0.06 ± 0.05	−0.06 ± 0.05^e^	−0.004 ± 0.014^d^	+0.07 ± 0.05	+0.04 ± 0.04	−0.03 ± 0.03
ICS × TIME	+0.004 ± 0.007	−0.017 ± 0.014	+0.037 ± 0.019	−0.004 ± 0.014	−0.010 ± 0.013	−0.008 ± 0.013	+0.002 ± 0.009
	(*N* = 1,665, *k* = 1.7)	(*N* = 428, *k* = 1.7)	(*N* = 330, *k* = 1.7)	(*N* = 514, *k* = 1.7)	(*N* = 393, *k* = 1.7)	(*N* = 729, *k* = 1.7)	(*N* = 936, *k* = 1.7)
**TRAILMAKING TEST, PART A**							
Intercept	+**35.3 ± 4.1**^b^	+**35.2 ± 4.1**^b^	+**42.6 ± 8.6**^b^	+**34.1 ± 21.5**^b^	+**37.6 ± 11.7**^b^	+**20.2 ± 2.8**^b^	+**47.9 ± 7.8**^b^
TIME	+2.32 ± 1.24	+**23.42 ± 7.04**^b^	+1.30 ± 2.60	−4.93 ± 6.56	+1.95 ± 3.98	+1.03 ± 0.66	+2.93 ± 2.20
ICS	+0.01 ± 0.74	−0.78 ± 1.77	−1.42 ± 1.54	+0.64 ± 1.05	+0.24 ± 1.54	−0.23 ± 0.42	+0.23 ± 0.36
ICS × TIME	+0.142 ± 0.230	+0.865 ± 0.582	+0.277 ± 0.511	+0.001 ± 0.314	−0.139 ± 0.483	−0.067 ± 0.106	+0.232 ± 0.364
	(*N* = 1,644, *k* = 1.7)	(*N* = 428, *k* = 1.7)	(*N* = 319, *k* = 1.6)	(*N* = 511, *k* = 1.7)	(*N* = 639, *k* = 1.7)	(*N* = 719, *k* = 1.7)	(*N* = 925, *k* = 1.7)
**TRAILMAKING TEST, PART B**							
Intercept	+**193.3 ± 35.1** ^b^	+217.5 ± 203.9	+**882.1 ± 188.5**^b^	+170.2 ± 86.7	+**181.5 ± 60.7**^b^	+106.5 ± 63.1	+**292.7 ± 28.0**^b^
TIME	+12.0 ± 10.9	+75.9 ± 48.0	−81.4 ± 44.4	+28.2 ± 26.2	+20.6 ± 11.2	+6.8 ± 16.8	+7.57 ± 7.92
ICS	+4.16 ± 2.78	−2.00 ± 5.65	+**17.00 ± 6.65**^b^	+0.46 ± 5.0	+2.32 ± 4.81	+0.08 ± 3.90	+**8.09 ± 3.78**^b^
ICS × TIME	−0.389 ± 0.608	+2.159 ± 1.365	−0.926 ± 1.725	+0.363 ± 1.136	−0.322 ± 0.711	−0.538 ± 0.787	+0.686 ± 0.851
	(*N* = 1,634, *k* = 1.6)	(*N* = 425, *k* = 1.6)	(*N* = 316, *k* = 1.6)	(*N* = 510, *k* = 1.7)	(*N* = 383, *k* = 1.6)	(*N* = 715, *k* = 1.6)	(*N* = 919, *k* = 1.6)

*CES-D, Center for Epidemiologic Studies-Depression; ICS, Inflammation composite score; MMSE, Mini-Mental State Examination; NSAIDs, Non-steroidal anti-inflammatory drugs; PIR, poverty income ratio; WRAT, Wide Range Achievement Test*.

a *Most cognitive test scores were in the direction of higher score = better performance, except for BVRT (total errors), and Trailmaking Test both parts (expressed in seconds). Models were controlled for: age (centered at 50 y), sex, race, poverty status, education, marital status, literacy, current smoking status, current drug use, body mass index (BMI, centered at 30), CES-D total score (centered at 15), HEI-2010 (centered at 40), self-reported diabetes, hypertension, high cholesterol, cardiovascular disease, inflammatory conditions, NSAIDs, and the inverse mills ratio. All covariates were interacted with TIME. All inverse mills ratios were centered at zero, except for DS-B, Trails A and B for whom the inverse mills ratio was centered at its mean*.

b P < 0.05 for null hypothesis that γ = 0;

c *P < 0.009 for null hypothesis that γ = 0 for interaction between ICS and TIME*.

d *p < 0.05 for null hypothesis of no by sex and Age group, based on 3-way and 4-way interaction terms with ICS and TIME*.

e *p < 0.05 for null hypothesis of no by race, based on 2-way and 3-way interaction terms with ICS and TIME*.

**Figure 1:**
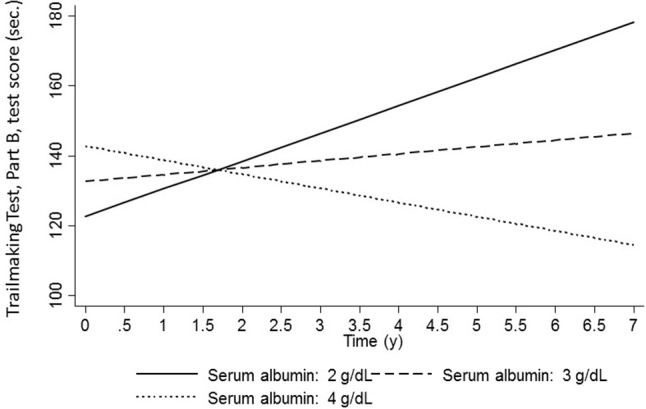
Predictive margins for Trailmaking test B (sec,) by serum albumin levels (g/dL): mixed-effects regression models: total population.

Examining individual markers of inflammation (Table S2), multiple-testing adjusted results suggested CRP was associated with poorer baseline mental status among younger women (≤50y, γ_01_ = −0.03 ± 0.01*, p* = 0.002) and poorer attention among older women (>50y, γ_01_ = −0.024 ± 0.007*, p* < 0.001) and African-Americans (γ_01_ = −0.029 ± 0.008*, p* = 0.001). Nevertheless, CRP was directly associated with an improvement in the same test of attention over time among the African-American group (γ_01_ = +0.006 ± 0.002*, p* = 0.002). For ESR as the main exposure (Table S3), there was a faster decline in verbal memory among older men (>50y, γ_11_ = −0.008 ± 0.003*, P* = 0.009); with poorer baseline performance on tests of attention overall (γ_01_ = −0.010 ± 0.003*, P* = 0.003) and among African-Americans (γ_01_ = −0.013 ± 0.004*, P* = 0.002); on a test of verbal fluency among older women (>50y, γ_01_ = −0.037 ± 0.013*, P* = 0.004) and on a test of executive function, overall (γ_01_ = +0.62 ± 0.21*, P* = 0.004), among older men (>50y, γ_01_ = +1.69 ± 0.53*, P* = 0.001) and among African-Americans (γ_01_ = +0.84 ± 0.28*, P* = 0.002). The latter association among others was race-specific (*P* < 0.05 for interaction between ESR and race), though no heterogeneity was detected by age and sex. Moreover, a higher baseline serum albumin as an individual marker was linked to slower attention decline among older men (>50y, γ_11_ = +0.329 ± 0.103*, P* = 0.009), improvement in executive function in the total population (γ_11_ = −6.00 ± 2.26*, P* = 0.008), and a better baseline performance in psychomotor speed among African-Americans (γ_01_ = +0.56 ± 0.19*, P* = 0.003). There were no significant associations between serum iron and cognitive outcomes. (Table S4) None of the serum iron key associations with cognitive performance, cross-sectional or longitudinal, remained significant after correction for multiple testing (Table S5). The relationship between serum albumin and executive function (Trails B) in the total population is illustrated in Figure 1, using predictive margins from the mixed-effects regression model.

## Discussion

To our knowledge, this is the first study of the relationships of systemic inflammation with trajectories of cognitive performance in a large sample of bi-racial urban adults examining associations systematically across age, sex, and race groups. Among key findings, a composite score combining 4 markers of systemic inflammation was associated with faster decline on a test of visual memory/visuo-constructive abilities, among older men only (>50y). Many other associations were detected in the expected direction for all markers except for serum iron, whereby a higher inflammatory status was linked to either worse performance at baseline or faster decline over time for specific age, sex and race groups. Most notably, baseline ESR was associated with a faster decline on verbal memory among older men (>50y), whereas serum albumin was linked to slower attention decline among older men (>50y) and over-time improvement in executive function in the total population. In contrast, hs-CRP's associations with cognition were mostly detected at baseline, for global mental status and the domain of attention.

Previous studies have focused on individual markers rather than a composite measure for inflammation, mostly hs-CRP, including many of the recent investigations (Bettcher et al., 2012; Obasi et al., 2012; Trollor et al., 2012; Arfanakis et al., 2013; O'bryant et al., 2013; Yarchoan et al., 2013; Krogh et al., 2014; Lima et al., 2014; Metti et al., 2014; Windham et al., 2014; Goldstein et al., 2015; Matsushima et al., 2015; Palta et al., 2015; Gong et al., 2016; Tampubolon, 2016; Tegeler et al., 2016; Watanabe et al., 2016; Hsu et al., 2017; Lai et al., 2017; Walker et al., 2017; Corlier et al., 2018; Hajjar et al., 2018; Warren et al., 2018). In earlier studies conducted between 2003 and 2011, focus was mostly on cognitive performance and decline rather than brain imaging outcomes. In those studies, 18 of 28 selected original studies found a direct relationship between hs-CRP and cognitive performance at one point in time (Mangiafico et al., 2006; Roberts et al., 2009, 2010; Hoshi et al., 2010; Noble et al., 2010; O'bryant et al., 2010; Canon and Crimmins, 2011) or decline over time (or incident cognitive impairment) (Yaffe et al., 2003; Engelhart et al., 2004; Komulainen et al., 2007; Hoth et al., 2008; Locascio et al., 2008; Luciano et al., 2009; Marioni et al., 2009; Mooijaart et al., 2011). Our study corroborated the former finding more than the latter, particularly for global mental status and the domain of attention. In previous studies, when a large battery of cognitive tests was used, hs-CRP were adversely linked to domains of verbal memory (Komulainen et al., 2007; Mooijaart et al., 2011), attention (Hoth et al., 2008), psychomotor speed (Hoth et al., 2008; Canon and Crimmins, 2011; Mooijaart et al., 2011), executive function (Hoth et al., 2008; Wersching et al., 2010), and visuo-spatial function (Noble et al., 2010), With the exception of the Whitehall II study (Gimeno et al., 2008) most of those earlier studies were conducted on older adults with mean ages >60y at baseline, as opposed to studying inflammation during mid-life. Thus, our study adds to the body of evidence of a cross-sectional inverse relationship between hs-CRP levels and cognitive performance in mid-life.

Among the most recent studies conducted as of 2012, at least 10 of 23 have found an association between elevated hs-CRP and worse cognitive, functional and structural brain outcomes. For instance, after 14,180 person-years of follow-up (UK sample, Age≥75y), a study found that hs-CRP was associated with worse cognitive performance in the domain of episodic memory particularly among older individuals (Tampubolon, 2016). Similarly, in the Atherosclerosis Risk in Communities (ARIC) study, higher hs-CRP was linked to lower white matter microstructural integrity, particularly among African-Americans (Walker et al., 2017). Delving deeper into AD-relevant brain regions, a recent longitudinal study of 335 elderly subjects found that an elevated baseline hs-CRP predicted thinner regional cortex at year 9, and that CRP itself acted as a mediator in the inverse relationship between baseline metabolic risk and regional cortical thickness (Corlier et al., 2018).

Serum fibrinogen was directly associated with worse cognitive performance in several recent studies (Gabay and Kushner, 1999; Van Oijen et al., 2005; Luciano et al., 2009; Marioni et al., 2009; Wada et al., 2011; Tampubolon, 2016). For instance, in a longitudinal study of 2,312 men and women aged 50 to 80 years participating in the Aspirin for Asymptomatic Atherosclerosis Trial (mean follow-up: 5y), adjusting for baseline cognitive scores and other covariates, baseline fibrinogen predicted decline in several cognitive domains (excluding memory), a finding also observed for hs-CRP (Marioni et al., 2009). However, a large longitudinal study (*n* = 6,713, mean follow-up~5.3y) found only fibrinogen (and not hs-CRP) was positively associated with incident dementia, AD and vascular dementia, suggesting fibrinogen's effect on cognition may be hemostatic rather than inflammatory (Van Oijen et al., 2005). Our study indicated that ESR, a proxy measure of fibrinogen, was linked to faster decline on verbal memory among older men, but not in other age/sex or race groups.

Studies that examined the association of serum albumin or iron with cognitive outcomes, though less numerous, were compelling (Dik et al., 2005; Kuo et al., 2007; Vupputuri et al., 2008; Ng et al., 2009; Llewellyn et al., 2010; Onem et al., 2010; Taniguchi et al., 2014; Koyama et al., 2016; Murayama et al., 2017). Specifically, microalbinuria was linked to worse cognitive performance measured by the Digits Symbol Substitution Test (DSST) (tapping into psychomotor speed) in a cross-sectional study of a nationally representative sample of older adults (≥60y, *n* = 2,049) (Kuo et al., 2007). In a longitudinal study of 1,664 Chinese older adults, lower albumin tertile was associated with greater risk of cognitive impairment at baseline [low, odds ratio (OR) = 2.30, 95% CI = 1.31–4.03; medium, OR = 1.59, 95% CI = 0.88–2.88] vs. high (*P* for trend = 0.002); and with cognitive decline in longitudinal analyses: low, OR = 1.73, 95% CI = 1.18–2.55; medium, OR = 1.32, 95% CI = 0.89–1.95, vs. high (*P* for trend = 0.004). In cognitively unimpaired respondents at baseline (MMSE ≥ 24), similar associations with cognitive decline were observed (*P* for trends < 0.002) (Ng et al., 2009). Our study indicated that the putative protective effect of serum albumin on cognition was relevant the total population as well as specific age/sex and race groups wereby a higher baseline serum albumin was linked to slower attention decline among older men improvement in executive function in the total population, and a better baseline performance in psychomotor speed among African-Americans. The latter finding is in line with the cross-sectional study previously described (Kuo et al., 2007). Our findings also highlight the putative protective effect of serum albumin on multiple domains of cognition.

Finally, two large Japanese cohort studies of older adults concluded that baseline serum albumin and baseline measures of iron status (e.g., hemoglobin) were independently associated with cognitive decline over varying follow-up periods (Taniguchi et al., 2014; Murayama et al., 2017). The finding for iron status was not replicated in our study. Moreover, our findings regarding a composite measure for the 4 inflammatory markers indicated that simultaneous increase in hs-CRP and ESR coupled with decreases in serum albumin and iron would lead to a faster decline on the dmain of visual memory/visuo-spatial ability among older men. This finding is novel and suggests biological interactions between inflammatory markers to influence the cognitive trajectory of older men in the domain of visual memroy. High CRP, low albumin and high ESR each had a specific link to cognitive decline for specific groups within this urban adult population. Thus, one cannot generalize to the entire population except for a few instances where Albumin and ESR had an association with cognitive performance and decline in the total sample, specifically in the domain of executive function (i.e. Trails B).

Both acute and chronic events leading to increased systemic inflammation induced by a variety of stimuli reportedly lead to microglia priming, increased production of proinflammatory molecules in the brain, and acceleration of cognitive decline in AD (Holmes et al., 2009; Van Eldik et al., 2016). The hepatic synthesis of acute-phase proteins, such as hs-CRP, is an exquisitely sensitive systemic marker of inflammation, infection, and tissue damage. As reported in our study, increased levels of hs-CRP are found in patients with newly diagnosed AD regardless of age of onset vs. healthy controls suggesting that an amplified neuroinflammatory reaction plays an important role in the pathogenesis and progression of neurocognitive decline in AD (Song et al., 2015). Low serum albumin levels acting as a negative acute phase reactant, reflect decreased liver function in the elderly and predispose to decreased antioxidant levels which may accelerate cognitive decline in this population (Soriani et al., 1994; Mizrahi et al., 2008; Llewellyn et al., 2010). Chronic anemia and abnormal iron-associated metabolism are well established risk factors for incident AD (Faux et al., 2014) and accelerated cognitive decline (Shah et al., 2011). A number of mechanisms may be at play including decreased plasma iron due to abnormal transferrin desaturation (Hare et al., 2015) and increased levels of oxidized hemoglobin and heme which abnormally bind and colocalize with amyloid-beta senile plaques causing cerebral amyloid angiopathy (Perry et al., 2008; Chuang et al., 2012).

Our study has several notable strengths, including adequate statistical power due to large sample size which allowed stratification by key socio-demographic factors. With two waves of cognitive data, a longitudinal design was possible thus ascertaining temporality of associations. Furthermore, an extensive cognitive battery was administered during those two waves, which enhanced our ability to study a variety of cognitive domains. We also used advanced techniques to adjust for both potential confounders and sample selectivity, while considering sampling weights for baseline covariates in our descriptive analyses. Finally, to study overall effect of systemic inflammation, principal components analysis was conducted to obtain a composite measure based on 4 individual markers.

Nevertheless, our findings are tempered by several limitations. First, despite adjustment for most key confounders, we cannot rule out residual confounding. Second, outcome-related limitations include having only 2-time points when cognitive performance was measured, limited decline over time possibly due to young age of the cohort, inability to create domains due to lack of factorial invariance across key socio-demographic factors. These limitations and others are detailed in previous studies (e.g., Beydoun et al., 2018). Although use of NSAIDs and chronic inflammatory conditions were accounted for in our study, acute inflammation due to injury was not readily available, though it is assumed to be a rare event in our population. In addition, inconsistent findings in terms of cross-sectional and longitudinal relationships between CRP and attention among African-Americans should be further investigated. Moreover, it would be ideal to study these relationships across ApoE4 status, as was done in previous studies. However, genotype data was only available on a sub-sample of African-American HANDLS participants which precluded this type of analysis (Lima et al., 2014; Watanabe et al., 2016).

In sum, there were strong longitudinal and cross-sectional associations between systemic inflammation and cognitive performance, largely among older individuals (>50y) and African-Americans. Future randomized trials should monitor systemic markers of inflammation and cognitive performance over time.

## Author contributions

MB: conceptualization, plan of analysis, literature search and review, data management, statistical analysis, write-up of the manuscript, revision of the manuscript; GD and HB: Literature search and review, write-up of parts of the manuscript, revision of the manuscript; J-AC and HL: Literature review, write-up of parts of the manuscript, revision of manuscript; ME: Data acquisition, write-up of parts of the manuscript, revision of the manuscript; AZ: Data acquisition, plan of analysis, write-up of parts of the manuscript, revision of the manuscript.

### Conflict of interest statement

The authors declare that the research was conducted in the absence of any commercial or financial relationships that could be construed as a potential conflict of interest.

## Abbreviations

AD: Alzheimer's Disease
AF: Animal Fluency test
ALB: Albumin
BTA: Brief Test of Attention
BVRT: Benton Visual Retention Test
CDT: Clock Drawing Test
CES-D: Center for Epidemiologic Studies-Depression
CVLT-DFR: California Verbal Learning Test, Delayed Free Recal; (List A)
CVLT-List A: California Verbal Learning Test, immediate recall (List A)
CRP: C-reactive protein
DS-B: Digit Span Backwards
DS-F: Digit Span Forward
HANDLS: Healthy Aging in Neighborhoods of Diversity Across the Life Span
hs-CRP: High sensitivity C-reactive protein
HS: High School
IL: Interleukin
OLS: Ordinary Least Square
PIR: Poverty Income Ratio
Trails A: Trailmaking test, Part A
Trails B: Trailmaking test, Part B
WBC: White Blood Cells
WRAT: Wide Range Achievement Test.
