# Supplementary material for: Systemic Inflammation Is Associated With Longitudinal Changes in Cognitive Performance Among Urban Adults
Source: Front Aging Neurosci. 2018 Oct 9;10:313. doi: 10.3389/fnagi.2018.00313 (PMC6189312; doi:10.3389/fnagi.2018.00313)
Supplement: Supplementary file 1 [file Table_1.DOCX]

**Table S1.** Cognitive performance test scores at baseline (Visit 1), follow-up (Visit 2), and change between visits, by age group/sex and by race, for HANDLS participants with complete and reliable baseline and/or follow-up cognitive scores^a^

|  | **All** | **Older women**  **(>50y)** | **Older men**  **(>50y)** | **Younger women**  **(≤50y)** | **Younger men**  **(≤50y)** | **Whites** | **African-Americans** |
| --- | --- | --- | --- | --- | --- | --- | --- |
| *Mini-Mental State Exam, total score* |  |  |  |  |  |  |  |
| Visit 1 | 27.9±0.1 | 27.8±0.2 | **27.3±0.2 ^b^** | 28.1±0.1 | 28.0±0.1 | **28.5±0.1 ^c^** | 27.5±0.1 |
|  | *(N=2,574)* | *(N=668)* | *(N=511)* | *(N=792)* | *(N=603)* | *(N=1,107)* | *(N=1,467)* |
| Visit 2 | 28.0±0.1 | 28.0±0.1 | **27.6±0.2 ^b^** | 28.2±0.1 | 28.2±0.1 | **28.6±0.1 ^c^** | 27.7±0.1 |
|  | *(N=1,934)* | *(N=506)* | *(N=341)* | *(N=653)* | *(N=434)* | *(N=767)* | *(N=1,167)* |
| P (Visit2-Visit1) | *0.07* | *0.47* | *0.29* | *0.46* | *0.41* | *0.047* | *0.21* |
| *California Verbal Learning Test (CVLT), List A* |  |  |  |  |  |  |  |
| Visit 1 | 25.1±0.3 | 25.1±0.4 | **22.7±0.4 ^b^** | **27.1±0.5 ^b^** | 24.2±0.6 | **27.0±0.4 ^c^** | 24.0±0.4 |
|  | *(N=2,124)* | *(N=548)* | *(N=415)* | *(N=660)* | *(N=501)* | *(N=885)* | *(N=1,239)* |
| Visit 2 | 20.1±0.3 | 20.0±0.4 | **16.5±0.5 ^b^** | **21.9±0.5 ^b^** | 20.2±0.5 | **22.5±0.4 ^c^** | 18.7±0.3 |
|  | *(N=1,976)* | *(N=509)* | *(N=358)* | *(N=650)* | *(N=459)* | *(N=781)* | *(N=1,195)* |
| P (Visit2-Visit1) | *<0.001* | *<0.001* | *<0.001* | *<0.001* | *<0.001* | *<0.001* | *<0.001* |
| *CVLT, free delayed recall* |  |  |  |  |  |  |  |
| Visit 1 | 7.4±0.1 | 7.1±0.2 | **6.4±0.2 ^b^** | **8.2±0.2 ^b^** | 7.2±0.3 | **8.4±0.2 ^c^** | 6.8±0.2 |
|  | *(N=2,044)* | *(N=529)* | *(N=404)* | *(N=636)* | *(N=475)* | *(N=853)* | *(N=1,191)* |
| Visit 2 | 5.8±0.1 | 5.7±0.2 | **4.2±0.3 ^b^** | 6.5±0.3 | 6.0±0.2 | **7.2±0.2 ^c^** | 5.1±0.2 |
|  | *(N=1,846)* | *(N=481)* | *(N=327)* | *(N=606)* | *(N=432)* | *(N=719)* | *(N=1,127)* |
| P (Visit2-Visit1) | *<0.001* | *<0.001* | *<0.001* | *<0.001* | *0.001* | *<0.001* | *<0.001* |
| *Benton Visual Retention Test* |  |  |  |  |  |  |  |
| Visit 1 | 5.6±0.2 | **6.6±0.4 ^b^** | **6.1±0.3 ^b^** | **5.5±0.3 ^b^** | 4.5±0.3 | **4.9±0.2 ^c^** | 6.0±0.2 |
|  | *(N=2,537)* | *(N=653)* | *(N=503)* | *(N=785)* | *(N=596)* | *(N=1,095)* | *(N=1,442)* |
| Visit 2 | 7.6±0.2 | **9.1±0.3 ^b^** | **8.9±0.4 ^b^** | **7.3±0.3 ^b^** | 6.1±0.3 | **6.2±0.2 ^c^** | 8.4±0.2 |
|  | *(N=2,085)* | *(N=532)* | *(N=382)* | *(N=692)* | *(N=479)* | *(N=816)* | *(N=1,269)* |
| P (Visit2-Visit1) | *<0.001* | *<0.001* | *<0.001* | *<0.001* | *<0.001* | *<0.001* | *<0.001* |
| *Brief Test of Attention* |  |  |  |  |  |  |  |
| Visit 1 | 6.8±0.1 | 6.6±0.2 | 6.5±0.2 | 7.0±0.2 | 6.7±0.2 | **7.5±0.1 ^c^** | 6.3±0.1 |
|  | *(N=2,147)* | *(N=547)* | *(N=436)* | *(N=666)* | *(N=498)* | *(N=911)* | *(N=1,236)* |
| Visit 2 | 6.6±0.1 | 6.6±0.1 | 6.3±0.2 | 6.8±0.2 | 6.7±0.2 | **7.2±0.1 ^c^** | 6.3±0.1 |
|  | *(N=1,907)* | *(N=486)* | *(N=347)* | *(N=632)* | *(N=442)* | *(N=772)* | *(N=1,135)* |
| P (Visit2-Visit1) | *0.38* | *0.90* | *0.38* | *0.28* | *0.81* | *0.027* | *0.96* |
| *Animal Fluency* |  |  |  |  |  |  |  |
| Visit 1 | 19.3±0.2 | **18.3±0.3 ^b^** | **19.0±0.3 ^b^** | **19.0±0.4 ^b^** | 20.5±0.4 | **21.4±0.3 ^c^** | 18.1±0.3 |
|  | *(N=2,577)* | *(N=665)* | *(N=520)* | *(N=793)* | *(N=599)* | *(N=1,109)* | *(N=1,468)* |
| Visit 2 | 19.5±0.2 | **18.5±0.4 ^b^** | **19.2±0.4 ^b^** | **19.3±0.4 ^b^** | 20.7±0.6 | **21.7±0.3 ^c^** | 18.3±0.3 |
|  | *(N=2,139)* | *(N=548)* | *(N=403)* | *(N=696)* | *(N=492)* | *(N=839)* | *(N=1,300)* |
| P (Visit2-Visit1) | *0.59* | *0.61* | *0.74* | *0.71* | *0.85* | *0.58* | *0.60* |
| *Digits Span, Forward* |  |  |  |  |  |  |  |
| Visit 1 | 7.4±0.1 | **7.1±0.1 ^b^** | 7.4±0.2 | 7.6±0.1 | 7.5±0.2 | **8.1±0.1 ^c^** | 7.1±0.1 |
|  | *(N=2,524)* | *(N=643)* | *(N=505)* | *(N=781)* | *(N=595)* | *(N=1,081)* | *(N=1,443)* |
| Visit 2 | 7.5±0.1 | **7.0±0.1 ^b^** | **7.2±0.2 ^b^** | 7.7±0.2 | 7.8±0.2 | **8.2±0.1 ^c^** | 7.1±0.1 |
|  | *(N=1,971)* | *(N=499)* | *(N=372)* | *(N=643)* | *(N=457)* | *(N=760)* | *(N=1,211)* |
| P (Visit2-Visit1) | *0.65* | *0.53* | *0.35* | *0.51* | *0.37* | *0.29* | *0.94* |
| *Digits Span, Backward* |  |  |  |  |  |  |  |
| Visit 1 | 5.8±0.1 | 5.7±0.1 | 5.6±0.2 | 5.9±0.1 | 5.9±0.2 | **6.7±0.1 ^c^** | 5.3±0.1 |
|  | *(N=2,505)* | *(N=635)* | *(N=501)* | *(N=777)* | *(N=592)* | *(N=1,079)* | *(N=1,426)* |
| Visit 2 | 5.8±0.1 | 5.6±0.2 | **5.4±0.2 ^b^** | 5.9±0.1 | 6.0±0.2 | **6.7±0.1 ^c^** | 5.3±0.1 |
|  | *(N=1,965)* | *(N=499)* | *(N=370)* | *(N=642)* | *(N=454)* | *(N=755)* | *(N=1,210)* |
| P (Visit2-Visit1) | *0.81* | *0.85* | *0.30* | *0.97* | *0.73* | *0.98* | *0.94* |
| *Clock, command* |  |  |  |  |  |  |  |
| Visit 1 | 8.8±0.0 | **8.6±0.1 ^b^** | 8.9±0.1 | 8.8±0.1 | 8.9±0.1 | **9.0±0.0 ^c^** | 8.7±0.1 |
|  | *(N=2,582)* | *(N=661)* | *(N=515)* | *(N=800)* | *(N=606)* | *(N=1,117)* | *(N=1,465)* |
| Visit 2 | 8.8±0.0 | 8.7±0.1 | 8.7±0.1 | 8.8±0.1 | 8.9±0.1 | **9.0±0.1 ^c^** | 8.6±0.1 |
|  | *(N=2,104)* | *(N=539)* | *(N=386)* | *(N=692)* | *(N=487)* | *(N=829)* | *(N=1,275)* |
| P (Visit2-Visit1) | *0.82* | *0.34* | *0.19* | *0.73* | *0.90* | *0.91* | *0.93* |
| *Trailmaking test, Part A* |  |  |  |  |  |  |  |
| Visit 1 | 34.3±0.6 | **40.8±2.0^b^** | **38.2±1.0^b^** | 30.3±0.8 | 31.7±0.9 | **29.0±0.4 ^b^** | 37.5±0.9 |
|  | *(N=2,466)* | *(N=640)* | *(N=476)* | *(N=771)* | *(N=579)* | *(N=1,074)* | *(N=1,392)* |
| Visit 2 | 36.5±1.4 | 44.4±5.5 | **41.0±1.5^b^** | 30.9±0.8 | 34.7±2.5 | **29.9±0.7 ^b^** | 40.0±2.1 |
|  | *(N=1,874)* | *(N=492)* | *(N=339)* | *(N=619)* | *(N=424)* | *(N=774)* | *(N=1,100)* |
| P (Visit2-Visit1) | *0.15* | *0.54* | *0.54* | *0.61* | *0.25* | *0.29* | *0.26* |
| *Trailmaking test, Part B* |  |  |  |  |  |  |  |
|  |  |  |  |  |  |  |  |
| Visit 1 | 130.2±4.5 | **154.8±8.9 ^b^** | **154.8±10.3 ^b^** | 109.7±6.5 | 120.4±10.5 | **87.9±3.4 ^c^** | 155.3±6.7 |
|  | *(N=2,465)* | *(N=640)* | *(N=476)* | *(N=770)* | *(N=579)* | *(N=1,074)* | *(N=1,391)* |
| Visit 2 | 127.9±5.8 | 136.4±9.4 | **154.4±13.9 ^b^** | 120.2±10.8 | 114.2±11.5 | **77.2±2.3 ^c^** | 156.0±8.6 |
|  | *(N=1,728)* | *(N=445)* | *(N=306)* | *(N=578)* | *(N=399)* | *(N=724)* | *(N=1,004)* |
| P (Visit2-Visit1) | *0.75* | *0.16* | *0.98* | *0.41* | *0.69* | *0.009* | *0.96* |

*Key*: CES-D=Center for Epidemiologic Studies-Depression; MMSE=Mini-Mental State Examination; PIR=poverty income ratio; WRAT=Wide Range Achievement Test.

^a^ Most cognitive test scores were in the direction of higher score=better performance, except for BVRT (total errors), and Trailmaking Test both parts (expressed in seconds).

^b^ p<0.05 for null hypothesis of no difference in means of cognitive test scores by sex and Age group within each visit (referent category: Younger men). Wald test from svy:reg command.

^c^ p<0.05 for null hypothesis of no difference in means of cognitive test scores by race within each visit (referent category: Whites). Wald test from svy:reg command.

**Table S2.** Cognitive performance test scores by C-reactive protein (CRP), stratified by age group/sex and by race, for HANDLS participants with complete and reliable baseline and/or follow-up cognitive scores: mixed-effects regression models^a^

|  | **All** | **Older women**  **(>50y)** | **Older men**  **(>50y)** | **Younger women**  **(≤50y)** | **Younger men**  **(≤50y)** | **Whites** | **African-**  **Americans** |
| --- | --- | --- | --- | --- | --- | --- | --- |
| *Mini-Mental State Exam, total score* |  |  |  |  |  |  |  |
| Intercept | **+26.7±0.2^b^** | **+27.9±0.4^b^** | **+25.7±0.5 ^b^** | **+27.2±0.3 ^b^** | **+25.7±0.6^b^** | **+27.1±0.2^b^** | **+26.2±0.3^b^** |
| TIME | +0.12±0.05 **^b^** | +0.02±0.12 | +0.22±0.15 | +0.08±0.09 | +0.19±0.14 | +0.09±0.07 | +0.13±0.08 |
| CRP | +0.01±0.00 | +0.02±0.01 | +0.02±0.01 | **-0.03±0.01 ^b,c,e^** | -0.01±0.01 | +0.01±0.01 | +0.01±0.01 |
| CRP×TIME | -0.002±0.002 | -0.004±0.003 | -0.004±0.007 | +0.003±0.002 | +0.000±0.003 | **-0.006±0.003 ^b,f^** | +0.001±0.002 |
|  | *(N=1,705; k=1.7)* | *(N=439, k=1.7)* | *(N=340, k=1.6)* | *(N=531, k=1.7)* | *(N=395, k=1.6)* | *(N=746, k=1.6)* | *(N=959, k=1.7)* |
| *California Verbal Learning Test (CVLT), List A* |  |  |  |  |  |  |  |
| Intercept | **+24.6±0.7^b^** | **+24.9±1.4^b^** | **+21.2±1.4^b^** | **+23.6±3.1^b^** | **+21.4±2.0^b^** | **+25.3±1.0^b^** | **+22.2±1.0^b^** |
| TIME | **-1.38±0.17^b^** | **-1.30±0.37^b^** | **-1.83±0.34^b^** | **-1.74±0.75 ^b^** | -0.96±0.58 | **-1.54±0.26^b^** | **-1.08±0.22^b^** |
| CRP | -0.00±0.02 | -0.02±0.03 | +0.03±0.03 | -0.02±0.04 | +0.05±0.04 | +0.00±0.03 | -0.00±0.02 |
| CRP×TIME | +0.006±0.005 | +0.016±0.009 | -0.007±0.018 | +0.007±0.010 | -0.005±0.009 | **+0.023±0.011 ^b^** | +0.003±0.005 |
|  | *(N=1,633, k=1.6)* | *(N=420, k=1.6)* | *(N=323, k=1.5)* | *(N=516, k=1.6)* | *(N=374, k=1.6)* | *(N=710, k=1.6)* | *(N=923, k=1.6)* |
| *CVLT, free delayed recall* |  |  |  |  |  |  |  |
| Intercept | **+7.7±0.3^b^** | **+7.3±0.8^b^** | **+5.9±0.6^b^** | **+7.7±0.6^b^** | **+6.8±0.9 ^b^** | **+7.6±0.5^b^** | **+6.9±0.5^b^** |
| TIME | **-0.46±0.08^b^** | **-0.58±0.18^b^** | **-0.42±0.16^b^** | **-0.45±0.14^b^** | **-0.70±0.27 ^b^** | **-0.41±0.12 ^b^** | **-0.46±0.11^b^** |
| CRP | -0.01±0.01 | -0.01±0.02 | +0.02±0.01 | -0.02±0.02 | -0.04±0.03 | -0.005±0.017 | -0.01±0.01 |
| CRP×TIME | +0.002±0.002 | +0.005±0.004 | -0.011±0.008 | +0.001±0.005 | +0.007±0.005 | +0.008±0.005 | +0.001±0.003 |
|  | *(N=1,600, k=1.5)* | *(N=414, k=1.6)* | *(N=310, k=1.5)* | *(N=510, k=1.6)* | *(N=366, k=1.5)* | *(N=690, k=1.5)* | *(N=910, k=1.6)* |
| *Benton Visual Retention Test* |  |  |  |  |  |  |  |
| Intercept | **+9.1±0.5^b^** | **+9.5±1.1^b^** | **+9.3±1.1^b^** | **+7.9±0.9 ^b^** | **+6.7±1.3 ^b^** | **+7.9±0.6^b^** | **+10.2±0.8^b^** |
| TIME | **+0.35±0.13^b^** | +0.13±0.32 | +0.28±0.28 | **+0.45±0.20 ^b^** | **+0.89±0.33 ^b^** | **+0.34±0.16 ^b^** | **+0.66±0.19^b^** |
| CRP | +0.01±0.01 | +0.02±0.03 | +0.00±0.02 | **+0.06±0.03 ^b,e^** | **-0.04±0.02 ^b^** | +0.03±0.02 | +0.00±0.01 |
| CRP×TIME | +0.001±0.003 | -0.011±0.008 | -0.003±0.013 | +0.003±0.006 | +0.007±0.004 | -0.002±0.006 | +0.002±0.004 |
|  | *(N=1,710, k=1.7)* | *(N=438, k=1.7)* | *(N=341, k=1.6)* | *(N=533, k=1.7)* | *(N=398, k=1.7)* | *(N=749, k=1.7)* | *(N=961, k=1.7)* |
| *Brief Test of Attention* |  |  |  |  |  |  |  |
| Intercept | **+6.6±0.2^b^** | **+7.0±0.5^b^** | **+6.5±0.5^b^** | **+6.7±0.5^b^** | **+5.6±0.7^b^** | **+6.8±0.3^b^** | **+5.9±0.4^b^** |
| TIME | -0.10±0.06 | **-0.19±0.13 ^b^** | -0.03±0.15 | -0.02±0.11 | +0.02±0.19 | -0.13±0.09 | -0.05±0.09 |
| CRP | **-0.024±0.007 ^b,c^** | **-0.047±0.012 ^b,c^** | -0.02±0.02 | -0.00±0.01 | -0.02±0.01 | -0.02±0.01 | **-0.029±0.008 ^b,c^** |
| CRP×TIME | **+0.004±0.002 ^b^** | +0.001±0.003 | +0.011±0.007 | +0.000±0.003 | **+0.006±0.003 ^b^** | -0.001±0.004 | **+0.006±0.002 ^b,d^** |
|  | *(N=1,647, k=1.7)* | *(N=419, k=1.6)* | *(N=329, k=1.6)* | *(N=514, k=1.6)* | *(N=385, k=1.6)* | *(N=716, k=1.6)* | *(N=931, k=1.6)* |
| *Animal Fluency* |  |  |  |  |  |  |  |
| Intercept | **+17.5±0.6^b^** | **+17.6±1.1^b^** | **+15.5±1.2^b^** | **+17.7±1.0^b^** | **+19.0±1.7^b^** | **+17.0±0.8^b^** | **+16.6±0.8^b^** |
| TIME | -0.06±0.12 | **+0.41±0.25^b^** | +0.31±0.27 | -0.08±0.21 | **-0.78±0.38^b^** | +0.17±0.19 | -0.23±0.16 |
| CRP | -0.00±0.01 | +0.02±0.03 | -0.01±0.02 | +0.04±0.03 | -0.04±0.04 | **+0.07±0.03 ^b,^** **^f^** | **-0.03±0.02 ^b^** |
| CRP×TIME | +0.006±0.003 | +0.002±0.006 | +0.013±0.012 | +0.011±0.006 | +0.006±0.007 | +0.008±0.007 | +0.006±0.004 |
|  | *(N=1,717, k=1.7)* | *(N=440, k=1.7)* | *(N=347, k=1.7)* | *(N=533, k=1.7)* | *(N=397, k=1.7)* | *(N=750, k=1.7)* | *(N=967, k=1.7)* |
| *Digits Span, Forward* |  |  |  |  |  |  |  |
| Intercept | **+6.8±0.2^b^** | **+6.6±0.4^b^** | **+6.8±0.5^b^** | **+6.7±0.4^b^** | **+6.7±0.4^b^** | **+6.8±0.3^b^** | **+6.6±0.3^b^** |
| TIME | -0.00±0.05 | +0.04±0.10 | +0.04±0.11 | -0.15±0.10 | -0.00±0.16 | -0.02±0.08 | -0.03±0.06 |
| CRP | -0.00±0.01 | -0.01±0.01 | -0.01±0.01 | -0.01±0.01 | -0.00±0.01 | +0.01±0.01 | -0.01±0.01 |
| CRP×TIME | +0.000±0.001 | +0.001±0.003 | -0.005±0.005 | +0.000±0.003 | +0.001±0.002 | -0.003±0.002 | +0.001±0.001 |
|  | *(N=1,710, k=1.6)* | *(N=436, k=1.7)* | *(N=342, k=1.6)* | *(N=534, k=1.7)* | *(N=398, k=1.6)* | *(N=743, k=1.6)* | *(N=967, k=1.7)* |
| *Digits Span, Backward* |  |  |  |  |  |  |  |
| Intercept | +0.5±4.9 | +26.0±14.5 | +5.5±19.0 | +5.1±10.7 | -12.9±14.2 | -1.0±7.7 | +2.8±6.6 |
| TIME | +0.26±1.20 | -4.70 ±4.40 | -2.30±4.90 | +1.90±2.40 | +4.60±3.10 | -0.47±1.95 | +0.12±1.65 |
| CRP | -0.00±0.00 | +0.01±0.01 | -0.01±0.01 | -0.01±0.01 | -0.00±0.01 | -0.00±0.011 | -0.00±0.01 |
| CRP×TIME | -0.002±0.001 | -0.005±0.003 | -0.002±0.005 | -0.001±0.003 | -0.001±0.002 | -0.002±0.003 | -0.002±0.001 |
|  | *(N=1,712, k=1.6)* | *(N=436, k=1.6)* | *(N=342, k=1.6)* | *(N=535, k=1.7)* | *(N=399, k=1.6)* | *(N=745, k=1.6)* | *(N=967, k=1.7)* |
| *Clock, command* |  |  |  |  |  |  |  |
| Intercept | **+8.76±0.13 ^b^** | **+8.76±0.29^b^** | **+8.75±0.27^b^** | **+9.00±0.24^b^** | **+8.60±0.39 ^b^** | **+9.04±0.18 ^b^** | **+8.27±0.20 ^b^** |
| TIME | -0.05±0.04 | -0.12±0.08 | -0.02±0.09 | -0.08±0.07 | +0.03±0.12 | -0.05±0.06 | -0.03±0.05 |
| CRP | -0.00±0.00 | +0.00±0.01 | **-0.02±0.01^b,e^** | **-0.02±0.01 ^b,e^** | +0.01±0.01 | +0.00±0.01 | -0.01±0.00 |
| CRP×TIME | +0.001±0.001 | +0.001±0.002 | +0.000±0.004 | +0.003±0.002 | -0.001±0.001 | +0.001±0.002 | +0.001±0.001 |
|  | *(N=1,712, k=1.7)* | *(N=438, k=1.7)* | *(N=338, k=1.7)* | *(N=535, k=1.7)* | *(N=401, k=1.7)* | *(N=751, k=1.7)* | *(N=961, k=1.7)* |
| *Trailmaking test, Part A* |  |  |  |  |  |  |  |
| Intercept | **+35.7±4.0 ^b^** | **-51.6±22.8 ^b^** | **+43.6±8.3 ^b^** | **+43.6±8.3 ^b^** | **+37.2±11.5 ^b^** | **+21.2±2.7 ^b^** | **+47.6±7.7 ^b^** |
| TIME | +2.1±1.2 | **+24.2±6.9 ^b^** | +0.76±2.48 | -5.29±6.34 | +1.99±3.92 | +0.81±0.65 | +2.84±2.17 |
| CRP | -0.02±0.10 | -0.04±0.24 | -0.13±0.23 | +0.03±0.15 | -0.05±0.17 | +0.01±0.06 | -0.04±0.15 |
| CRP×TIME | +0.018±0.029 | +0.092±0.084 | -0.132±0.109 | +0.002±0.043 | -0.005±0.047 | -0.019±0.016 | +0.030±0.042 |
|  | *(N=1,691, k=1.7)* | *(N=438, k=1.7)* | *(N=327, k=1.6)* | *(N=532, k=1.7)* | *(N=394, k=1.7)* | *(N=741, k=1.7)* | *(N=950, k=1.7)* |
| *Trailmaking test, Part B* |  |  |  |  |  |  |  |
| Intercept | +189.1±34.6 | +182.4±203.6 | **+825.6±185.4 ^b^** | +162.6±84.3 | **+177.9±59.9^b^** | +111.7±61.7 | **+291.9±28.0 ^b^** |
| TIME | +11.1±10.6 | +87.3±47.6 | -82.1±43.6 | +26.5±25.3 | +19.6±11.0 | +6.6±16.2 | +8.3±7.9 |
| CRP | +0.16±0.37 | -0.02±0.79 | +1.83±1.00 | -0.26±0.71 | -0.36±0.54 | +0.50±0.58 | +0.06±0.48 |
| CRP×TIME | +0.016±0.074 | +0.229±0.195 | -0.297±0.377 | -+0.072±0.152 | -0.008±0.069 | -0.177±0.117 | +0.067±0.097 |
|  | *(N=1,680, k=1.6)* | *(N=435, k=1.6)* | *(N=324, k=1.6)* | *(N=530, k=1.7)* | *(N=391, k=1.6)* | *(N=737, k=1.6)* | *(N=943, k=1.6)* |

*Key*: CES-D=Center for Epidemiologic Studies-Depression; CRP=C-reactive protein; MMSE=Mini-Mental State Examination; NSAIDs=Non-steroidal anti-inflammatory drugs; PIR=poverty income ratio; WRAT=Wide Range Achievement Test.

^a^ Most cognitive test scores were in the direction of higher score=better performance, except for BVRT (total errors), and Trailmaking Test both parts (expressed in seconds). CRP was centered at 5. Models were controlled for: age (centered at 50y), sex, race, poverty status, education, marital status, literacy, current smoking status, current drug use, body mass index (BMI, centered at 30), CES-D total score (centered at 15), HEI-2010 (centered at 40), self-reported diabetes, hypertension, high cholesterol, cardiovascular disease, inflammatory conditions, NSAIDs and the inverse mills ratio. All covariates were interacted with TIME. All inverse mills ratios were centered at zero, except for DS-B, Trails A and B for whom the inverse mills ratio was centered at its mean.

^b^ P<0.05 for null hypothesis that γ=0; ^c^ P<0.004 for null hypothesis that γ=0 for main effect CRP; ^d^ P<0.009 for null hypothesis that γ=0 for interaction between CRP and TIME. ^e^ p<0.05 for null hypothesis of no by sex and Age group, based on 3-way and 4-way interaction terms with CRP and TIME.

^f^ p<0.05 for null hypothesis of no by race, based on 2-way and 3-way interaction terms with CRP and TIME.

**Table S3.** Cognitive performance test scores by Erythrocyte Sedimentation Rate (ESR), stratified by age group/sex and by race, for HANDLS participants with complete and reliable baseline and/or follow-up cognitive scores: mixed-effects regression models^a^

|  | **All** | **Older women**  **(>50y)** | **Older men**  **(>50y)** | **Younger women**  **(≤50y)** | **Younger men**  **(≤50y)** | **Whites** | **African-**  **Americans** |
| --- | --- | --- | --- | --- | --- | --- | --- |
| *Mini-Mental State Exam, total score* |  |  |  |  |  |  |  |
| Intercept | **+26.7±0.2^b^** | **+28.0±0.4^b^** | **+25.6±0.5 ^b^** | **+27.1±0.3 ^b^** | **+25.5±0.6^b^** | **+27.0±0.3^b^** | **+26.2±0.3^b^** |
| TIME | +0.11±0.06 | -0.01±0.12 | +0.21±0.14 | +0.07±0.09 | +0.21±0.15 | +0.10±0.08 | +0.10±0.08 |
| ESR | -0.00±0.00 | -0.00±0.00 | +0.00±0.01 | -0.00±0.00 | +0.01±0.01 | +0.00±0.01 | -0.00±0.00 ^f^ |
| ESR×TIME | +0.000±0.001 | -0.000±0.001 | +0.000±0.002 | +0.000±0.001 | -0.001±0.002 | -0.001±0.001 | +0.001±0.001 |
|  | *(N=1,680; k=1.7)* | *(N=437, k=1.7)* | *(N=337, k=1.6)* | *(N=517, k=1.7)* | *(N=389, k=1.6)* | *(N=729, k=1.6)* | *(N=951, k=1.7)* |
| *California Verbal Learning Test (CVLT), List A* |  |  |  |  |  |  |  |
| Intercept | **+26.6±0.2^b^** | **+25.2±1.4^b^** | **+25.2±1.4^b^** | **+22.7±3.3 ^b^** | **+21.4±2.0^b^** | **+25.4±1.1^b^** | **+22.2±1.0^b^** |
| TIME | **-1.36±0.17^b^** | **-1.24±0.38^b^** | **-1.86±0.34^b^** | **-1.85±0.33^b^** | -0.90±0.59 | **-1.68±0.27^b^** | **-1.01±0.22^b^** |
| ESR | -0.01±0.01 | -0.03±0.02 | -0.00±0.02 | -0.00±0.02 | +0.01±0.02 | -0.01±0.02 | -0.01±0.01 ^f^ |
| ESR×TIME | -0.001±0.002 | +0.003±0.005 | -0.012±0.006 | +0.003±0.004 | -0.004±0.005 | -0.007±0.005 | -0.003±0.003 ^f^ |
|  | *(N=1,608, k=1.6)* | *(N=418, k=1.6)* | *(N=320, k=1.5)* | *(N=503, k=1.6)* | *(N=367, k=1.6)* | *(N=694, k=1.5)* | *(N=914, k=1.6)* |
| *CVLT, free delayed recall* |  |  |  |  |  |  |  |
| Intercept | **+7.9±0.3^b^** | **+7.5±0.8^b^** | **+5.9±0.6^b^** | **+7.8±0.7^b^** | **+7.0±1.0^b^** | **+7.8±0.5^b^** | **+7.0±0.5^b^** |
| TIME | **-0.46±0.08^b^** | **-0.58±0.18^b^** | **-0.44±0.16^b^** | **-0.46±0.15^b^** | **-0.72±0.28 ^b^** | **-0.47±0.13 ^b^** | **-0.44±0.11^b^** |
| ESR | **-0.010±0.005 ^b^** | **-0.021±0.008 ^b^** | +0.00±0.01 | -0.01±0.01 | -0.01±0.01 | -0.00±0.01 | **-0.012±0.005 ^b^** |
| ESR×TIME | -0.000±0.001 | +0.002±0.002 | **-0.008±0.003^b,d^** | -0.001±0.002 | +0.001±0.003 | +0.002±0.002 | -0.001±0.001 |
|  | *(N=1,574, k=1.5)* | *(N=412, k=1.6)* | *(N=307, k=1.5)* | *(N=496, k=1.6)* | *(N=359, k=1.5)* | *(N=674, k=1.5)* | *(N=900, k=1.6)* |
| *Benton Visual Retention Test* |  |  |  |  |  |  |  |
| Intercept | **+9.2±0.5^b^** | **+9.6±1.1^b^** | **+9.3±1.1^b^** | **+7.7±1.0 ^b^** | **+7.6±1.0 ^b^** | **+8.1±0.7^b^** | **+10.2±0.8^b^** |
| TIME | **+0.33±0.13^b^** | +0.18±0.33 | +0.37±0.28 | **+0.45±0.21 ^b^** | **+0.86±0.34^b^** | **+0.36±0.17 ^b^** | **+0.59±0.19^b^** |
| ESR | -0.00±0.01 | -0.02±0.01 | +0.00±0.02 | +0.00±0.01 ^e^ | -0.00±0.02 | +0.00±0.00 | +0.00±0.01 |
| ESR×TIME | +0.004±0.002 | +0.001±0.004 | **+0.012±0.005 ^b^** | +0.002±0.003 | +0.002±0.004 | -0.000±0.003 | +0.005±0.002 |
|  | *(N=1,684, k=1.7)* | *(N=436, k=1.7)* | *(N=338, k=1.6)* | *(N=519, k=1.7)* | *(N=391, k=1.7)* | *(N=732, k=1.7)* | *(N=952, k=1.7)* |
| *Brief Test of Attention* |  |  |  |  |  |  |  |
| Intercept | **+6.8±0.2^b^** | **+7.0±0.5^b^** | **+6.3±0.5^b^** | **+6.9±0.5^b^** | **+5.8±0.7^b^** | **+6.8±0.3^b^** | **+6.1±0.4^b^** |
| TIME | **-0.13±0.06 ^b^** | -0.18±0.13 | -0.05±0.15 | -0.07±0.11 | -0.04±0.20 | -0.15±0.09 | -0.09±0.09 |
| ESR | **-0.010±0.003 ^b,c^** | -0.01±0.01 | -0.00±0.01 | +0.01±0.11 | -0.01±0.01 | -0.00±0.01 | **-0.013±0.004 ^b,c,f^** |
| ESR×TIME | +0.002±0.001 | +0.002±0.002 | -0.001±0.002 | +0.003±0.002 | -0.001±0.002 | +0.001±0.002 | +0.002±0.001 |
|  | *(N=1,622, k=1.6)* | *(N=417, k=1.6)* | *(N=326, k=1.5)* | *(N=501, k=1.6)* | *(N=378, k=1.6)* | *(N=700, k=1.6)* | *(N=922, k=1.6)* |
| *Animal Fluency* |  |  |  |  |  |  |  |
| Intercept | **+17.8±0.5^b^** | **+17.9±1.1^b^** | **+15.4±1.2^b^** | **+18.5±1.10 ^b^** | **+19.4±1.7^b^** | **+17.5±0.8^b^** | **+16.8±0.8^b^** |
| TIME | -0.07±0.12 | +0.35±0.25 | +0.28±0.28 | -0.07±0.22 | **-0.81±0.39 ^b^** | +0.07±0.20 | -0.21±0.16 |
| ESR | **-0.022±0.008 ^b^** | **-0.037±0.013^b,c^** | +0.01±0.02 | **-0.032±0.015 ^b^** | -0.02±0.02 | -0.02±0.02 | **-0.020±0.009 ^b^** |
| ESR×TIME | -0.000±0.002 | +0.005±0.002 | -0.007±0.004 | -0.000±0.003 | +0.000±0.004 | +0.005±0.004 | -0.001±0.002 |
|  | *(N=1,691, k=1.7)* | *(N=438, k=1.7)* | *(N=344, k=1.7)* | *(N=519, k=1.7)* | *(N=390, k=1.7)* | *(N=733, k=1.7)* | *(N=958, k=1.7)* |
| *Digits Span, Forward* |  |  |  |  |  |  |  |
| Intercept | **+6.9±0.2^b^** | **+6.7±0.4^b^** | **+6.8±0.5^b^** | **+6.6±0.5^b^** | **+7.7±0.7^b^** | **+6.9±0.3^b^** | **+6.6±0.3^b^** |
| TIME | +0.01±0.05 | +0.05±0.10 | +0.04±0.11 | -0.15±0.10 | -0.00±0.16 | +0.01±0.09 | -0.03±0.06 |
| ESR | -0.00±0.03 | +0.00±0.05^e^ | -0.01±0.01 | +0.00±0.01 | -0.01±0.01 | -0.00±0.01 | -0.00±0.00 |
| ESR×TIME | -0.000±0.001 | -0.001±0.001 | -0.001±0.002 | -0.000±0.001 | +0.000±0.002 | -0.002±0.002 | +0.000±0.003 |
|  | *(N=1,684, k=1.6)* | *(N=434, k=1.7)* | *(N=339, k=1.6)* | *(N=520, k=1.7)* | *(N=391, k=1.6)* | *(N=726, k=1.6)* | *(N=958, k=1.7)* |
| *Digits Span, Backward* |  |  |  |  |  |  |  |
| Intercept | +1.00±4.9 | +23.7±15.7 | +9.9±18.5 | +8.5±10.8 | -8.1±14.4 | +0.66±7.86 | +1.73±6.66 |
| TIME | -0.34±1.21 | -4.76 ±4.44 | -3.3±4.9 | +0.84±2.44 | +3.77±3.11 | -1.01±1.95 | -0.65±1.67 |
| ESR | -0.00±0.00 | +0.00±0.00 | -0.01±0.01 | -0.00±0.01 | **-0.02±0.01 ^b,e^** | +0.00±0.01 | -0.005±0.003 |
| ESR×TIME | -0.000±0.001 | -0.001±0.001 | -0.001±0.002 | -0.000±0.001 | +0.000±0.001 | -0.001±0.002 | -0.000±0.001 |
|  | *(N=1,686, k=1.6)* | *(N=434, k=1.7)* | *(N=339, k=1.6)* | *(N=521, k=1.7)* | *(N=392, k=1.6)* | *(N=728, k=1.6)* | *(N=958, k=1.7)* |
| *Clock, command* |  |  |  |  |  |  |  |
| Intercept | **+8.77±0.14 ^b^** | **+8.80±0.29^b^** | **+8.69±0.28^b^** | **+9.03±0.25 ^b^** | **+8.65±0.39 ^b^** | **+9.00±0.19 ^b^** | **+8.32±0.20 ^b^** |
| TIME | -0.06±0.04 | -0.15±0.08 | -0.01±0.09 | -0.07±0.07 | +0.03±0.12 | -0.06±0.06 | +0.04±0.05 |
| ESR | -0.00±0.00 | -0.00±0.00 | **-0.009±0.004 ^b^** | -0.00±0.00 | -0.00±0.00 | +0.00±0.00 | **-0.005±0.02 ^b,f^** |
| ESR×TIME | -0.000±0.001 | +0.001±0.001 | **+0.004±0.002 ^b^** | -0.001±0.001 | -0.001±0.001 | -0.001±0.001 | +0.000±0.001 |
|  | *(N=1,686, k=1.7)* | *(N=436, k=1.7)* | *(N=335, k=1.7)* | *(N=521, k=1.7)* | *(N=394, k=1.7)* | *(N=734, k=1.7)* | *(N=952, k=1.7)* |
| *Trailmaking test, Part A* |  |  |  |  |  |  |  |
| Intercept | **+34.9±4.1 ^b^** | **-51.8±22.6 ^b^** | **+43.5±8.5 ^b^** | +35.0±21.2 | **+37.0±11.8 ^b^** | **+20.1±2.8^b^** | **+47.3±7.9 ^b^** |
| TIME | +2.08±1.25 | **+21.5±6.9 ^b^** | +1.05±2.6 | -4.49±6.45 | +2.14±3.98 | +1.06±0.67 | +2.67±2.21 |
| ESR | +0.01±0.06 | -0.00±0.12 | -0.06±0.12 | +0.01±0.07 | +0.03±0.14 | +0.00±0.04 | -0.03±0.09 |
| ESR×TIME | +0.013±0.017 | +0.069±0.040 | +0.005±0.042 | -0.003±0.021 | -0.008±0.045 | -0.009±0.009 | +0.019±0.026 |
|  | *(N=1,664, k=1.7)* | *(N=436, k=1.7)* | *(N=323, k=1.6)* | *(N=518, k=1.7)* | *(N=xxx, k=1.7)* | *(N=723, k=1.7)* | *(N=941, k=1.7)* |
| *Trailmaking test, Part B* |  |  |  |  |  |  |  |
| Intercept | **+188±35.1 ^b^** | **+209±204 ^b^** | **+876±187^b^** | +170±87 | **+173.4±60.3^b^** | +103.1±63.0 | **+281.2±28.4^b^** |
| TIME | **+3.08±1.46 ^b^** | +0.13±0.40 | -79.3±43.3 | +29.7±26.9 | +21.1±11.1 | +8.45±16.68 | **+7.72±8.10** |
| ESR | **+0.62±0.21 ^b,c^** | +0.13±0.40 | **+1.69±0.53 ^b,c^** | +0.32±0.36 | +0.85±0.43 | +0.23±0.32 | **+0.84±0.28 ^b,c^** |
| ESR×TIME | +0.015±0.048 | +0.124±0.094 | -0.075±0.153 | -0.020±0.080 | -0.044±0.069 | -0.076±0.069 | +0.043±0.064 |
|  | *(N=1,654, k=1.6)* | *(N=443, k=1.6)* | *(N=320, k=1.6)* | *(N=517, k=1.7)* | *(N=384, k=1.6)* | *(N=719, k=1.6)* | *(N=935, k=1.6)* |

*Key*: CES-D=Center for Epidemiologic Studies-Depression; ESR= Erythrocyte Sedimentation Rate; MMSE=Mini-Mental State Examination; NSAIDs=Non-steroidal anti-inflammatory drugs; PIR=poverty income ratio; WRAT=Wide Range Achievement Test.

^a^ Most cognitive test scores were in the direction of higher score=better performance, except for BVRT (total errors), and Trailmaking Test both parts (expressed in seconds). ESR was centered at 16. Models were controlled for: age (centered at 50y), sex, race, poverty status, education, marital status, literacy, current smoking status, current drug use, body mass index (BMI, centered at 30), CES-D total score (centered at 15), HEI-2010 (centered at 40), self-reported diabetes, hypertension, high cholesterol, cardiovascular disease, inflammatory conditions, NSAIDs and the inverse mills ratio. All covariates were interacted with TIME. All inverse mills ratios were centered at zero, except for DS-B, Trails A and B for whom the inverse mills ratio was centered at its mean.

^b^ P<0.05 for null hypothesis that γ=0; ^c^ P<0.004 for null hypothesis that γ=0 for main effect ESR; ^d^ P<0.009 for null hypothesis that γ=0 for interaction between ESR and TIME. ^e^ p<0.05 for null hypothesis of no by sex and Age group, based on 3-way and 4-way interaction terms with ESR and TIME.

^f^ p<0.05 for null hypothesis of no by race, based on 2-way and 3-way interaction terms with ESR and TIME.

**Table S4.** Cognitive performance test scores by serum albumin, stratified by age group/sex and by race, for HANDLS participants with complete and reliable baseline and/or follow-up cognitive scores: mixed-effects regression models^a^

|  | **All** | **Older women**  **(>50y)** | **Older men**  **(>50y)** | **Younger women**  **(≤50y)** | **Younger men**  **(≤50y)** | **Whites** | **African-**  **Americans** |
| --- | --- | --- | --- | --- | --- | --- | --- |
| *Mini-Mental State Exam, total score* |  |  |  |  |  |  |  |
| Intercept | **+26.6±0.2^b^** | **+28.0±0.4^b^** | **+25.3±0.5 ^b^** | **+27.3±0.4 ^b^** | **+25.7±0.6^b^** | **+27.0±0.3^b^** | **+26.0±0.3^b^** |
| TIME | **+0.14±0.06 ^b^** | +0.03±0.13 | +0.30±0.16 | +0.13±0.10 | +0.16±0.15 | +0.08±0.09 | +0.17±0.09 |
| ALBUMIN | -0.15±0.13 | +0.08±0.28 | -0.67±0.35 | +0.11±0.22 | -0.08±0.25 | -0.04±0.19 | -0.22±0.18 |
| ALBUMIN×TIME | +0.041±0.036 | +0.018±0.08 | +0.146±0.100 | +0.068±0.064 | -0.060±0.063 | +0.027±0.058 | +0.054±0.048 |
|  | *(N=1,707; k=1.7)* | *(N=440, k=1.7)* | *(N=339, k=1.6)* | *(N=533, k=1.7)* | *(N=395, k=1.6)* | *(N=749, k=1.6)* | *(N=958, k=1.7)* |
| *California Verbal Learning Test (CVLT), List A* |  |  |  |  |  |  |  |
| Intercept | **+24.9±0.8^b^** | **+24.8±1.6^b^** | **+21.0±1.5^b^** | **+25.3±3.2^b^** | **+21.3±2.0^b^** | **+25.8±1.2^b^** | **+22.4±1.1^b^** |
| TIME | **-1.42±0.19^b^** | **-1.40±0.43^b^** | **-1.88±0.38^b^** | **-1.95±0.77 ^b^** | -0.96±0.59 | **-1.82±0.31^b^** | **-1.07±0.24^b^** |
| ALBUMIN | +0.22±0.49 | -0.17±1.02 | -0.00±1.00 | +1.78±0.96 ^e^ | -0.26±0.89 | +0.46±0.83 | +0.27±0.60 |
| ALBUMIN×TIME | -0.063±0.121 | -0.211±0.272 | -0.084±0.256 | -0.228±0.227 | -0.015±0.231 | -0.288±0.217 | +0.027±0.146 |
|  | *(N=1,635, k=1.6)* | *(N=421, k=1.6)* | *(N=322, k=1.5)* | *(N=518, k=1.6)* | *(N=374, k=1.6)* | *(N=713, k=1.5)* | *(N=922, k=1.6)* |
| *CVLT, free delayed recall* |  |  |  |  |  |  |  |
| Intercept | **+8.0±0.4^b^** | **+7.1±0.8^b^** | **+5.8±0.7^b^** | **+8.8±0.7^b^** | **+6.9±1.0^b^** | **+8.0±0.6^b^** | **+7.1±0.5^b^** |
| TIME | **-0.50±0.09^b^** | **-0.71±0.20^b^** | **-0.41±0.18^b^** | **-0.48±0.16^b^** | **-0.75±0.28 ^b^** | **-0.43±0.15 ^b^** | **-0.51±0.12^b^** |
| ALBUMIN | +0.31±0.23 | -0.23±0.49 | +0.05±0.46 | **+1.23±0.44 ^b,^** ^e^ | +0.40±0.42 | +0.35±0.40 | +0.31±0.28 |
| ALBUMIN×TIME | -0.050±0.058 | -0.188±0.119 | +0.030±0.120 | -0.023±0.110 | -0.118±0.124 | +0.008±0.106 | -0.072±0.070 |
|  | *(N=1,602, k=1.5)* | *(N=415, k=1.6)* | *(N=309, k=1.5)* | *(N=512, k=1.6)* | *(N=366, k=1.5)* | *(N=693, k=1.5)* | *(N=909, k=1.6)* |
| *Benton Visual Retention Test* |  |  |  |  |  |  |  |
| Intercept | **+9.3±0.6^b^** | **+10.3±1.3^b^** | **+9.4±1.2^b^** | **+8.1±1.1^b^** | **+7.0±1.3 ^b^** | **+7.76±0.7^b^** | **+10.7±0.9^b^** |
| TIME | +0.22±0.14 | -0.11±0.37 | +0.02±0.31 | +0.29±0.23 | **+0.91±0.34^b^** | +0.26±0.19 | **+0.45±0.21^b^** |
| ALBUMIN | +0.26±0.36 | +1.29±0.79 | +0.54±0.83 | -0.20±0.65 | +0.46±0.61 | -0.32±0.52 | +0.75±0.49 |
| ALBUMIN×TIME | +0.181±0.089 | -0.357±0.229 | -0.432±0.205 | -0.204±0.147 | -0.075±0.151 | -0.094±0.134 | **-0.291±0.118 ^b^** |
|  | *(N=1,712, k=1.7)* | *(N=439, k=1.7)* | *(N=340, k=1.6)* | *(N=535, k=1.7)* | *(N=398, k=1.7)* | *(N=752, k=1.7)* | *(N=960, k=1.7)* |
| *Brief Test of Attention* |  |  |  |  |  |  |  |
| Intercept | **+6.5±0.3^b^** | **+6.9±0.6^b^** | **+6.1±0.6^b^** | **+6.8±0.5^b^** | **+5.5±0.7^b^** | **+6.8±0.4^b^** | **+5.8±0.4^b^** |
| TIME | -0.03±0.07 | -0.15±0.15 | +0.15±0.16 | +0.04±0.12 | +0.01±0.19 | -0.06±0.10 | +0.02±0.10 |
| ALBUMIN | -0.09±0.16 | -0.06±0.36 | -0.55±0.37 | +0.16±0.32 | -0.14±0.29 | -0.02±0.26 | -0.13±0.22 |
| ALBUMIN×TIME | **+0.101±0.043 ^b^** | -0.005±0.091 | **+0.329±0.103^b,d,e^** | +0.088±0.081 | +0.017±0.084 | +0.081±0.073 | **+0.109±0.054 ^b^** |
|  | *(N=1,651, k=1.6)* | *(N=421, k=1.6)* | *(N=329, k=1.5)* | *(N=516, k=1.6)* | *(N=385, k=1.6)* | *(N=719, k=1.6)* | *(N=932, k=1.6)* |
| *Animal Fluency* |  |  |  |  |  |  |  |
| Intercept | **+18.0±0.6^b^** | **+17.7±1.2^b^** | **+15.3±1.3^b^** | **+18.7±1.2^b^** | **+19.5±1.7 ^b^** | **17.6±0.9^b^** | **+16.9±0.8^b^** |
| TIME | -0.00±0.13 | +0.48±0.28 | +0.34±0.29 | -0.04±0.24 | -0.70±0.39 | +0.01±0.23 | -0.06±0.17 |
| ALBUMIN | +0.61±0.38 | +0.07±0.77 | -0.27±0.86 | +0.84±0.72 | +0.87±0.75 | +0.47±0.64 | +0.58±0.47 |
| ALBUMIN×TIME | +0101±0.084 | +0.122±0.168 | +0.057±0.190 | +0.070±0.155 | +0.202±0.176 | -0.146±0.158 | **+0.229±0.098^b^** |
|  | *(N=1,719, k=1.7)* | *(N=441, k=1.7)* | *(N=346, k=1.7)* | *(N=535, k=1.7)* | *(N=397, k=1.7)* | *(N=753, k=1.7)* | *(N=966, k=1.7)* |
| *Digits Span, Forward* |  |  |  |  |  |  |  |
| Intercept | **+7.2±0.2^b^** | **+7.0±0.5^b^** | **+7.1±0.5^b^** | **+6.8±0.5^b^** | **+7.8±0.7^b^** | **+7.0±0.4^b^** | **+7.0±0.3^b^** |
| TIME | -0.01±0.06 | +0.02±0.11 | -0.00±0.12 | -0.18±0.11 | +0.01±0.16 | -0.01±0.10 | -0.06±0.07 |
| ALBUMIN | **+0.41±0.15 ^b^** | +0.28±0.31 | +0.57±0.34 | +0.07±0.30 | +0.44±0.30 | +0.13±0.25 | **+0.56±0.19 ^b,c^** |
| ALBUMIN×TIME | -0.017±0.035 | -0.023±0.071 | -0.070±0.080 | -0.025±0.073 | +0.037±0.068 | +0.014±0.069 | -0.040±0.041 |
|  | *(N=1,712, k=1.6)* | *(N=437, k=1.7)* | *(N=341, k=1.6)* | *(N=536, k=1.7)* | *(N=398, k=1.6)* | *(N=746, k=1.6)* | *(N=966, k=1.7)* |
| *Digits Span, Backward* |  |  |  |  |  |  |  |
| Intercept | +0.2±4.9 | +22.8±15.3 | +3.3±19.0 | +6.4±10.7 | -12.9±14.3 | -0.4±7.7 | +1.5±6.6 |
| TIME | +0.62±1.20 | -2.8 ±4.4 | -1.6±4.8 | +1.7±2.4 | +4.6±3.1 | -0.37±1.93 | +0.64±1.65 |
| ALBUMIN | +0.02±0.15 | -0.27±0.30 | -0.03±0.31 | +0.12±0.28 | +0.09±0.30 | -0.37±0.25 | +0.25±0.18 |
| ALBUMIN×TIME | +0.010±0.036 | -0.042±0.081 | +0.053±0.082 | -0.052±0.065 | +0.043±0.067 | +0.028±0.065 | -0.002±0.043 |
|  | *(N=1,714, k=1.6)* | *(N=437, k=1.6)* | *(N=341, k=1.6)* | *(N=537, k=1.7)* | *(N=399, k=1.6)* | *(N=748, k=1.6)* | *(N=966, k=1.7)* |
| *Clock, command* |  |  |  |  |  |  |  |
| Intercept | **+8.71±0.15 ^b^** | **+8.43±0.32^b^** | **+8.68±0.30^b^** | **+9.20±0.26 ^b^** | **+8.51±0.39 ^b^** | **+8.97±0.21 ^b^** | **+8.24±0.23 ^b^** |
| TIME | -0.04±0.04 | -0.06±0.09 | -0.01±0.10 | -0.12±0.07 | -0.04±0.12 | -0.06±0.07 | -0.00±0.58 |
| ALBUMIN | -0.08±0.09 | **-0.06±0.09 ^b^** | -0.07±0.20 | +0.22±0.16 | -0.20±0.18 | -0.12±0.14 | -0.02±0.12 |
| ALBUMIN×TIME | +0.032±0.027 | **+0.110±0.056 ^b^** | -0.006±0.066 | -0.048±0.048 | -0.037±0.051 | +0.013±0.045 | +0.036±0.033 |
|  | *(N=1,713, k=1.7)* | *(N=439, k=1.7)* | *(N=337, k=1.7)* | *(N=536, k=1.7)* | *(N=401, k=1.7)* | *(N=754, k=1.7)* | *(N=959, k=1.7)* |
| *Trailmaking test, Part A* |  |  |  |  |  |  |  |
| Intercept | **+37.2±4.4 ^b^** | -36.6±22.1 | **+47.1±8.9 ^b^** | **+40.1±20.7 ^b^** | **+37.0±11.8 ^b^** | **+22.4±2.9^b^** | **+49.3±8.4 ^b^** |
| TIME | +1.66±1.34 | **+18.38±6.72 ^b^** | +0.13±2.70 | -6.30±6.39 | +2.38±4.01 | +0.59±0.70 | +2.23±2.39 |
| ALBUMIN | +2.45±2.66 | +10.07±7.17 | +6.06±5.73 | -0.47±3.54 | -0.64±5.25 | +2.23±1.46 | +2.34±4.44 |
| ALBUMIN×TIME | -0.61±0.84 | -2.592±2.313 | -1.152±1.759 | -0.707±1.096 | +0.669±1.825 | -0.299±0.384 | -0.828±1.353 |
|  | *(N=1,693, k=1.7)* | *(N=439, k=1.7)* | *(N=326, k=1.6)* | *(N=534, k=1.7)* | *(N=394, k=1.7)* | *(N=744, k=1.7)* | *(N=949, k=1.7)* |
| *Trailmaking test, Part B* |  |  |  |  |  |  |  |
| Intercept | **+197.8±35.3 ^b^** | +211.9±202.5 | **+855.8±186.3^b^** | **+172.1±85.0 ^b^** | **+174.0±60.5^b^** | **+140.9±62.6 ^b^** | **+284.7±29.7^b^** |
| TIME | +6.22±10.71 | +69.3±47.1 | **-89.6±43.6 ^b^** | +25.7±25.1 | +18.9±11.0 | +1.96±16.3 | +1.9±8.3 |
| ALBUMIN | +10.18±10.01 | +25.3±23.1 | 16.2±24.9 | +21.3±16.9 | -7.5±16.5 | **+29.4±13.2 ^b,f^** | -10.4±14.2 |
| ALBUMIN×TIME | **-6.00±2.26 ^b,d^** | -3.619±5.553 | **-12.47±5.91 ^b^** | -7.321±4.001 | -2.12±2.74 | -3.288±2.910 | **-6.410±3.204 ^b^** |
|  | *(N=1,682, k=1.6)* | *(N=436, k=1.6)* | *(N=323, k=1.6)* | *(N=532, k=1.7)* | *(N=391, k=1.6)* | *(N=740, k=1.6)* | *(N=942, k=1.6)* |

*Key*: ALBUMIN= serum albumin; CES-D=Center for Epidemiologic Studies-Depression; MMSE=Mini-Mental State Examination; NSAIDs=Non-steroidal anti-inflammatory drugs; PIR=poverty income ratio; WRAT=Wide Range Achievement Test.

^a^ Most cognitive test scores were in the direction of higher score=better performance, except for BVRT (total errors), and Trailmaking Test both parts (expressed in seconds). ALBUMIN was centered at 4. Models were controlled for: age (centered at 50y), sex, race, poverty status, education, marital status, literacy, current smoking status, current drug use, body mass index (BMI, centered at 30), CES-D total score (centered at 15), HEI-2010 (centered at 40), self-reported diabetes, hypertension, high cholesterol, cardiovascular disease, inflammatory conditions, NSAIDs and the inverse mills ratio. All covariates were interacted with TIME. All inverse mills ratios were centered at zero, except for DS-B, Trails A and B for whom the inverse mills ratio was centered at its mean.

^b^ P<0.05 for null hypothesis that γ=0; ^c^ P<0.004 for null hypothesis that γ=0 for main effect ALBUMIN; ^d^ P<0.009 for null hypothesis that γ=0 for interaction between ALBUMIN and TIME. ^e^ p<0.05 for null hypothesis of no by sex and Age group, based on 3-way and 4-way interaction terms with ALBUMIN and TIME.

^f^ p<0.05 for null hypothesis of no by race, based on 2-way and 3-way interaction terms with ALBUMIN and TIME.

**Table S5.** Cognitive performance test scores by serum Iron, stratified by age group/sex and by race, for HANDLS participants with complete and reliable baseline and/or follow-up cognitive scores: mixed-effects regression models^a^

|  | **All** | **Older women**  **(>50y)** | **Older men**  **(>50y)** | **Younger women**  **(≤50y)** | **Younger men**  **(≤50y)** | **Whites** | **African-**  **Americans** |
| --- | --- | --- | --- | --- | --- | --- | --- |
| *Mini-Mental State Exam, total score* |  |  |  |  |  |  |  |
| Intercept | **+26.7±0.2^b^** | **+28.0±0.4^b^** | **+25.7±0.5 ^b^** | **+27.2±0.3 ^b^** | **+25.7±0.6^b^** | **+27.2±0.2^b^** | **+26.2±0.3^b^** |
| TIME | **+0.11±0.05 ^b^** | -0.02±0.12 | +0.22±0.15 | +0.08±0.09 | +0.19±0.14 | +0.06±0.07 | +0.12±0.08 |
| IRON | -0.00±0.00 | -0.00±0.0 | -0.00±0.00 | +0.00±0.00 | +0.00±0.00 | +0.00±0.00 | -0.00±0.00 |
| IRON×TIME | +0.000±0.000 | +0.001±0.001 | -0.000±0.001 | +0.000±0.000 | -0.000±0.001 | -0.000±0.000 | +0.000±0.004 |
|  | *(N=1,705; k=1.7)* | *(N=438, k=1.7)* | *(N=341, k=1.6)* | *(N=531, k=1.7)* | *(N=395, k=1.6)* | *(N=749, k=1.6)* | *(N=956, k=1.7)* |
| *California Verbal Learning Test (CVLT), List A* |  |  |  |  |  |  |  |
| Intercept | **+24.8±0.7^b^** | **+25.1±1.4^b^** | **+21.2±1.4^b^** | **+24.1±3.1^b^** | **+21.4±2.0^b^** | **+25.5±1.0^b^** | **+22.3±1.0^b^** |
| TIME | **-1.38±0.17^b^** | **-1.24±0.37^b^** | **-1.81±0.34^b^** | **-1.74±0.75 ^b^** | -0.98±0.58 | **-1.59±0.26^b^** | **-1.10±0.21^b^** |
| IRON | -0.00±0.00 | -0.01±0.01 | -0.00±0.01 | +0.01±0.01**^e^** | -0.01±0.01 | -0.01±0.01 | +0.00±0.01 |
| IRON×TIME | +0.001±0.001 | +0.001±0.002 | -0.001±0.002 | +0.001±0.001 | +0.001±0.002 | +0.000±0.001 | +0.001±0.001 |
|  | *(N=1,634, k=1.6)* | *(N=420, k=1.6)* | *(N=324, k=1.5)* | *(N=516, k=1.6)* | *(N=374, k=1.6)* | *(N=713, k=1.5)* | *(N=921, k=1.6)* |
| *CVLT, free delayed recall* |  |  |  |  |  |  |  |
| Intercept | **+7.8±0.3^b^** | **+7.4±0.8^b^** | **+6.1±0.6^b^** | **+7.9±0.6^b^** | **+6.8±1.0^b^** | **+7.8±0.5^b^** | **+6.9±0.5^b^** |
| TIME | **-0.47±0.08 ^b^** | **-0.56±0.18^b^** | **-0.43±0.16^b^** | **-0.46±0.14^b^** | **-0.71±0.27 ^b^** | **-0.44±0.12 ^b^** | **-0.46±0.11^b^** |
| IRON | -0.00±0.00 | -0.00±0.00 | +0.01±0.00 | **-0.01±0.00 ^b,e^** | -0.00±0.00 | -0.00±0.00 | -0.00±0.00 |
| IRON×TIME | -0.000±0.000 | -0.000±0.001 | +0.000±0.001 | -0.000±0.001 | +0.000±0.001 | +0.000±0.001 | -0.000±0.001 |
|  | *(N=1,601, k=1.5)* | *(N=414, k=1.6)* | *(N=311, k=1.5)* | *(N=510, k=1.6)* | *(N=366, k=1.5)* | *(N=693, k=1.5)* | *(N=908, k=1.6)* |
| *Benton Visual Retention Test* |  |  |  |  |  |  |  |
| Intercept | **+9.2±0.5^b^** | **+9.3±1.1^b^** | **+9.1±1.2^b^** | **+8.3±0.9^b^** | **+6.7±1.3 ^b^** | **+8.0±0.6^b^** | **+10.2±0.8^b^** |
| TIME | **+0.35±0.13^b^** | +0.20±0.33 | +0.30±0.28 | +0.44±0.20 | **+0.86±0.33^b^** | **+0.33±0.16 ^b^** | **+0.65±0.19^b^** |
| IRON | +0.00±0.00 | -0.01±0.01 | +0.00±0.01 | +0.00±0.00 | +0.01±0.01 | +0.00±0.00 | -0.00±0.00 |
| IRON×TIME | -0.000±0.000 | +0.002±0.002 | -0.001±0.001 | -0.000±0.001 | +0.001±0.001 | -0.001±0.001 | +0.000±0.001 |
|  | *(N=1,710, k=1.7)* | *(N=437, k=1.7)* | *(N=342, k=1.6)* | *(N=533, k=1.7)* | *(N=398, k=1.7)* | *(N=752, k=1.7)* | *(N=958, k=1.7)* |
| *Brief Test of Attention* |  |  |  |  |  |  |  |
| Intercept | **+6.6±0.2^b^** | **+7.0±0.5^b^** | **+6.4±0.6^b^** | **+6.7±0.5^b^** | **+5.6±0.7^b^** | **+6.8±0.3^b^** | **+5.9±0.4^b^** |
| TIME | -0.10±0.06 | -0.18±0.13 | -0.00±0.19 | -0.02±0.11 | -0.01±0.19 | -0.12±0.09 | -0.05±0.09 |
| IRON | -0.00±0.00 | +0.00±0.00 ^e^ | +0.00±0.00 | -0.00±0.00^e^ | **-0.01±0.00 ^b^** | +0.00±0.00 | -0.00±0.00 |
| IRON×TIME | +0.000±0.000 | +0.001±0.001 | -0.001±0.001 | +0.000±0.000 | -0.000±0.001 | +0.000±0.001 | -0.000±0.000 |
|  | *(N=1,649, k=1.6)* | *(N=420, k=1.6)* | *(N=330, k=1.6)* | *(N=514, k=1.6)* | *(N=385, k=1.6)* | *(N=719, k=1.6)* | *(N=930, k=1.6)* |
| *Animal Fluency* |  |  |  |  |  |  |  |
| Intercept | **+17.6±0.6^b^** | **+17.8±1.1^b^** | **+15.8±1.2^b^** | **+18.2±1.1^b^** | **+19.1±1.7^b^** | **17.3±0.8^b^** | **+16.5±0.8^b^** |
| TIME | -0.08±0.12 | +0.40±0.25 | +0.28±0.27 | **-**0.10±0.21 | **-0.80±0.38^b^** | +0.13±0.19 | -0.23±0.16 |
| IRON | +0.00±0.00 | -0.00±0.01 | **-0.013±0.006 ^b^** | **+0.011±0.005 ^b^** | -0.00±0.01 | +0.00±0.01 | -0.00±0.00 |
| IRON×TIME | +0.000±0.001 | **-0.004±0.002 ^b^** | +0.001±0.001 | -0.001±0.001 | +0.002±0.002 | +0.001±0.001 | -0.000±0.001 |
|  | *(N=1,717, k=1.7)* | *(N=439, k=1.7)* | *(N=348, k=1.7)* | *(N=533, k=1.7)* | *(N=397, k=1.7)* | *(N=753, k=1.7)* | *(N=964, k=1.7)* |
| *Digits Span, Forward* |  |  |  |  |  |  |  |
| Intercept | **+6.9±0.3^b^** | **+6.8±0.4^b^** | **+6.8±0.5^b^** | **+6.7±0.4^b^** | **+7.6±0.7^b^** | **+6.9±0.3^b^** | **+6.36±0.3^b^** |
| TIME | -0.01±0.05 | +0.05±0.10 | +0.05±0.11 | -0.16±0.10 | -0.00±0.16 | -0.02±0.08 | -0.03±0.06 |
| IRON | +0.00±0.00 | -0.00±0.00 | +0.000±0.002 | +0.00±0.00 | +0.00±0.00 | -0.00±0.00 | +0.00±0.01 |
| IRON×TIME | +0.000±0.000 | -0.000±0.001 | -0.000±0.001 | +0.001±0.000 | +0.000±0.001 | +0.001±0.000 | +0.000±0.000 |
|  | *(N=1,711, k=1.6)* | *(N=436, k=1.7)* | *(N=343, k=1.6)* | *(N=534, k=1.7)* | *(N=398, k=1.6)* | *(N=746, k=1.6)* | *(N=965, k=1.7)* |
| *Digits Span, Backward* |  |  |  |  |  |  |  |
| Intercept | +0.7±4.9 | +27.2±15.3 | 2.0±19.1 | +6.4±10.7 | -12.9±14.2 | -0.54±7.69 | +2.47±6.58 |
| TIME | +0.27±1.20 | -4.4 ±4.4 | -1.9±4.9 | +1.96±2.42 | +4.51±3.11 | -0.32±1.93 | +0.01±1.66 |
| IRON | -0.00±0.00 | **-0.006±0.002 ^b^** | -0.001±0.002 | +0.001±0.002 | -0.001±0.003 | -0.001±0.002 | -0.002±0.002 |
| IRON×TIME | +0.000±0.000 | +0.000±0.001 | +0.000±0.001 | +0.000±0.000 | +0.000±0.001 | +0.000±0.000 | -0.000±0.000 |
|  | *(N=1,713, k=1.6)* | *(N=436, k=1.6)* | *(N=343, k=1.6)* | *(N=535, k=1.7)* | *(N=399, k=1.6)* | *(N=748, k=1.6)* | *(N=965, k=1.7)* |
| *Clock, command* |  |  |  |  |  |  |  |
| Intercept | **+8.77±0.13 ^b^** | **+8.76±0.29^b^** | **+8.74±0.28^b^** | **+9.05±0.24 ^b^** | **+8.63±0.39 ^b^** | **+9.06±0.18 ^b^** | **+8.26±0.20 ^b^** |
| TIME | -0.06±0.04 | -0.15±0.08 | +0.00±0.09 | -0.07±0.07 | +0.02±0.12 | -0.06±0.06 | -0.03±0.05 |
| IRON | -0.00±0.00 | +0.00±0.00 | -0.00±0.00 | +0.00±0.00^e^ | **-0.004±0.002 ^b^** | +0.00±0.00 | -0.00±0.00 |
| IRON×TIME | +0.001±0.000 | +0.000±0.000 | -0.000±0.000 | +0.001±0.000 | +0.000±0.000 | +0.000±0.000 | +0.000±0.000 |
|  | *(N=1,712, k=1.7)* | *(N=437, k=1.7)* | *(N=339, k=1.7)* | *(N=535, k=1.7)* | *(N=401, k=1.7)* | *(N=754, k=1.7)* | *(N=958, k=1.7)* |
| *Trailmaking test, Part A* |  |  |  |  |  |  |  |
| Intercept | **+35.6±4.0 ^b^** | **-51.2±22.8 ^b^** | **+43.5±8.3 ^b^** | 38.1±20.9 | **+37.3±11.5 ^b^** | **+21.0±2.7^b^** | **+47.7±7.7 ^b^** |
| TIME | +2.05±1.21 | **+23.1±6.9 ^b^** | +0.68±2.50 | -5.1±6.3 | +2.06±3.92 | +0.80±0.65 | +2.79±2.18 |
| IRON | -0.02±0.02 | +0.01±0.06 | +0.01±0.04 | -0.03±0.03 | -0.02±0.05 | -0.00±0.01 | -0.03±0.04 |
| IRON×TIME | +0.002±0.006 | +0.002±0.019 | +0.004±0.011 | +0.002±0.007 | -0.001±0.018 | +0.001±0.002 | +0.004±0.010 |
|  | *(N=1,691, k=1.7)* | *(N=437, k=1.7)* | *(N=328, k=1.6)* | *(N=532, k=1.7)* | *(N=394, k=1.7)* | *(N=744, k=1.7)* | *(N=947, k=1.7)* |
| *Trailmaking test, Part B* |  |  |  |  |  |  |  |
| Intercept | **+189.7±34.5 ^b^** | +177.2±202.4 | **+833.8±184.5^b^** | **+171.5±84.2 ^b^** | **+177.2±59.9^b^** | +115.1±61.4 | +292.2±27.9**^b^** |
| TIME | +10.7±10.6 | +77.6±47.3 | -81.4±43.1 | +26.5±25.1 | +20.2±11.0 | +5.0±16.0 | +7.6±7.9 |
| IRON | -0.09±0.08 | +0.19±0.19 | -0.31±0.17 | -0.17±0.12 | +0.12±0.17 | -0.14±0.10 | -0.08±0.12 |
| IRON×TIME | +0.017±0.016 | -0.029±0.044 | +0.048±0.037 | +0.018±0.024 | +0.038±0.026 | +0.012±0.018 | +0.021±0.024 |
|  | *(N=1,680, k=1.6)* | *(N=434, k=1.6)* | *(N=325, k=1.6)* | *(N=530, k=1.7)* | *(N=391, k=1.6)* | *(N=740, k=1.6)* | *(N=940, k=1.6)* |

*Key*: CES-D=Center for Epidemiologic Studies-Depression; IRON= serum Iron; MMSE=Mini-Mental State Examination; NSAIDs=Non-steroidal anti-inflammatory drugs; PIR=poverty income ratio; WRAT=Wide Range Achievement Test.

^a^ Most cognitive test scores were in the direction of higher score=better performance, except for BVRT (total errors), and Trailmaking Test both parts (expressed in seconds). IRON was centered at 84. Models were controlled for: age (centered at 50y), race, poverty status, education, marital status, literacy, current smoking status, current drug use, body mass index (BMI, centered at 30), CES-D total score (centered at 15), HEI-2010 (centered at 40), self-reported diabetes, hypertension, high cholesterol, cardiovascular disease, inflammatory conditions, NSAIDs and the inverse mills ratio. All covariates were interacted with TIME. All inverse mills ratios were centered at zero, except for DS-B, Trails A and B for whom the inverse mills ratio was centered at its mean.

^b^ P<0.05 for null hypothesis that γ=0; ^c^ P<0.004 for null hypothesis that γ=0 for main effect IRON; ^d^ P<0.009 for null hypothesis that γ=0 for interaction between IRON and TIME. ^e^ p<0.05 for null hypothesis of no by sex and Age group, based on 3-way and 4-way interaction terms with IRON and TIME.

^f^ p<0.05 for null hypothesis of no by race, based on 2-way and 3-way interaction terms with IRON and TIME.
