# Supplementary material for: Systemic Inflammation Is Associated With Longitudinal Changes in Cognitive Performance Among Urban Adults
Source: Front Aging Neurosci. 2018 Oct 9;10:313. doi: 10.3389/fnagi.2018.00313 (PMC6189312; doi:10.3389/fnagi.2018.00313)
Supplement: Supplementary file 2 [file Data_Sheet_1.PDF]

Online Supporting Material

Supplemental method 1: Description of cognitive tests, literacy and the CES-D

Mini-Mental State Examination (MMSE)

The MMSE (1) is a brief mental status test and global cognitive functioning measuring orientation, concentration, immediate and delayed memory, language and constructional praxis. Scores range from 0 to 30, with higher scores indicating better cognitive performance.

California Verbal Learning Test (CVLT)

The CVLT (2) is a 16-item shopping list measuring verbal learning and memory. A modified version of the CVLT was used with three, rather than five, list A learning trials. Cued recall was not administered. Variables of interest in this study were total correct for List A sum across trials 1-3 and List A long-delay free recall. Scores ranged from 0 to 48 for List A sum and 0 to 16 for List A long-delay free recall. Higher scores indicate better verbal memory. The CVLT is described in detail elsewhere (2).

Benton Visual Retention Test (BVRT)

The BVRT (3) is a test of short-term figural memory and visuo-constructional abilities. Administration A, Form D was used. Two trained examiners independently scored the BVRT using a modified error scoring system, based on the BVRT Manual scoring. A consensus was achieved for discrepancies in scoring. If a consensus between the two examiners could not be reached, MKT, a research psychologist assigned the score. Scores were total errors, such that higher values indicate poorer visual memory.

Online Supporting Material

Digit Span Forward and Backward (DS-F and DS-B)

The Wechsler Adult Intelligence Scale, Revised(4) Digit Span Forward and Backward are tests of attention and executive functioning, specifically working memory. They were administered according to standard instructions, and the total score was the total number correct for each test.

Animal Fluency

Animal fluency, a measure of semantic verbal fluency, requires participants to generate as many animals as possible for 60 seconds. Higher scores indicate better verbal fluency, with the total number of words, minus intrusions and perseverations analyzed.

Brief Test of Attention (BTA)

The BTA (5) is a measure of divided auditory attention. An examiner administered 10 trials where increasing longer lists of letters and numbers (containing 4-18 items) were read. Participants were instructed to keep track of how many numbers were read during each trial, disregarding the number of letters, and were told to keep their hands in fists to discourage counting on their fingers. Only the numbers portion of the test was administered. The total score was the total number of trials correct out of 10.

Online Supporting Material

Trail Making Tests A and B (Trails A and Trails B)

Trailmaking test A and B(6) are tests of attention and executive functioning, respectively, specifically cognitive control and visuo-motor scanning/processing speed. Participants were instructed to draw lines between consecutive numbers (Trails A) or alternate between numbers and letter (Trails B) as fast as they could while a stop watch recorded time. When errors were committed the participant corrected the error by returning to his/her last correct response and continued from there. The stop-watch ran while corrections were made. Scores reflected time to completion (in seconds) separately for Trails A and B. Higher scores indicate poorer performance.

Clock Drawing Test – Clock to Command (CDT)

The Clock Drawing Test (7) is a test of visuo-spatial and visuo-constructional abilities. Participants are asked to draw a clock, put in all of the numbers and set the hands for 10 after 11. Scores are assessed for the clock face (0-2), numbers (0-4) and hands (0-4), with a range from 0 to 10, with higher scores indicating more accurate clock drawing. Participants who did not score a 10 on the command version of the test were asked to copy a clock with the time set to 10 after 11.

Wide Range Achievement Test – 3rd Edition: Word and Letter Reading Subtest (WRAT)

The WRAT Word and Letter Reading Subtest (8) is a test of verbal knowledge, frequently used as a proxy for literacy and educational quality. Participants were asked to pronounce a list of 50 words that increased in difficulty. If a criterion of the first five words correctly pronounced was not reached, letter reading was administered. The tan form was administered according to standard instruction and the score was the total number of words correctly pronounced.

Online Supporting Material

Center for Epidemiological Studies Depression Scale (CES-D)

The CES-D (9) is a 20-item measure of depressive symptoms. Participants are asked to rate the frequency and severity of symptoms over the past week. Scores range from 0 to 60, with scores of 16 and higher indicating significant depressive symptoms, and scores of 20 and higher indicating significant clinically depressive symptoms.

Supplemental Method 2: Description of mixed-effects regression models

The main multiple mixed-effects regression models can be summarized as follows:

Eq.	**Multi-level models**	vs.	**Composite models**
1.1-1.4	$Y_{ij} = \pi_{0i} + \pi_{1i} Time_{ij} + \varepsilon_{ij}$	$\pi_{0i} = \gamma_{00} + \gamma_{0a} X_{aij} + \sum_{k=1}^{l} \gamma_{0k} Z_{ik} + \zeta_{0i}$ $\pi_{1i} = \gamma_{10} + \gamma_{1a} X_{aij} + \sum_{m=1}^{n} \gamma_{1m} Z_{im} + \zeta_{1i}$	$Y_{ij} = \gamma_{00} + \gamma_{0a} X_{aij} + \sum_{k=1}^{l} \gamma_{0k} Z_{ik}$ $+ \gamma_{10} Time_{ij} + \gamma_{1a} X_{aij} Time_{ij}$ $+ \sum_{m=1}^{n} \gamma_{1m} Z_{im} Time_{ij}$ $+ (\zeta_{0i} + \zeta_{1i} Time_{ij} + \varepsilon_{ij})$

Where Y_{ij} is the outcome (cognitive test scores) for each individual "i" and visit "j"; π_{0i} is the level-1 intercept for individual i; π_{1i} is the level-1 slope for individual i; γ_{00} is the level-2 intercept of the random intercept π_{0i} ; γ_{10} is the level-2 intercept of the slope π_{1i} ; Z_{ik} is a vector of fixed covariates for each individual *i* that are used to predict level-1 intercepts and slopes and included baseline age (Age_{base}) among other covariates. $X_{ija,}$ represents the main predictor variables (one of the inflammation exposures); ζ_{0i} and ζ_{1i} are level-2 disturbances; ε_{ij} is the within-person level-1 disturbance. Of primary interest are the main effects of each exposure X_a (γ_{0a}) and their interaction with *TIME* (γ_{1a}), as described in a previous methodolgical paper.(10)

References

1. Folstein MF, Folstein SE, McHugh PR. "Mini-mental state". A practical method for grading the cognitive state of patients for the clinician. J Psychiatr Res 1975; 12:189-198
2. Delis DC, Freeland J, Kramer JH, Kaplan E. Integrating clinical assessment with cognitive neuroscience: construct validation of the California Verbal Learning Test. J Consult Clin Psychol 1988; 56:123-130
3. Benton AL, ed. Revised visual retention test (fifth edition). New York: The Psychological Corportation; 1974.
4. Wechsler D. WAIS-R manual. Cleveland: The Psychological Corporation;1981.
5. Schretlen D, Bobholz JH, Brandt J. Development and psychometric properties of the Brief Test of Attention. Clinical Neuropsychologist 1996; 10:80-89
6. Reitan R. Trail Making Test: Manual for Administration and Scoring. Tucson, AZ: Reitan Neuropsychological Laboratory.
7. Rouleau I, Salmon DP, Butters N, Kennedy C, McGuire K. QUANTITATIVE AND QUALITATIVE ANALYSES OF CLOCK DRAWINGS IN ALZHEIMERS AND HUNTINGTONS-DISEASE. Brain and Cognition 1992; 18:70-87
8. Wilkinson GS. Wide Range Achievement Test–Revision 3. Wilmington, DE: Jastak Association.
9. Nguyen HT, Kitner-Triolo M, Evans MK, Zonderman AB. Factorial invariance of the CES-D in low socioeconomic status African Americans compared with a nationally representative sample. Psychiatry research 2004; 126:177-187
10. Blackwell E, de Leon CF, Miller GE. Applying mixed regression models to the analysis of repeated-measures data in psychosomatic medicine. Psychosom Med 2006; 68:870-878
