# Supplementary figures and images for: Systemic Inflammation Is Associated With Longitudinal Changes in Cognitive Performance Among Urban Adults
Source: Front Aging Neurosci. 2018 Oct 9;10:313. doi: 10.3389/fnagi.2018.00313 (PMC6189312; doi:10.3389/fnagi.2018.00313)

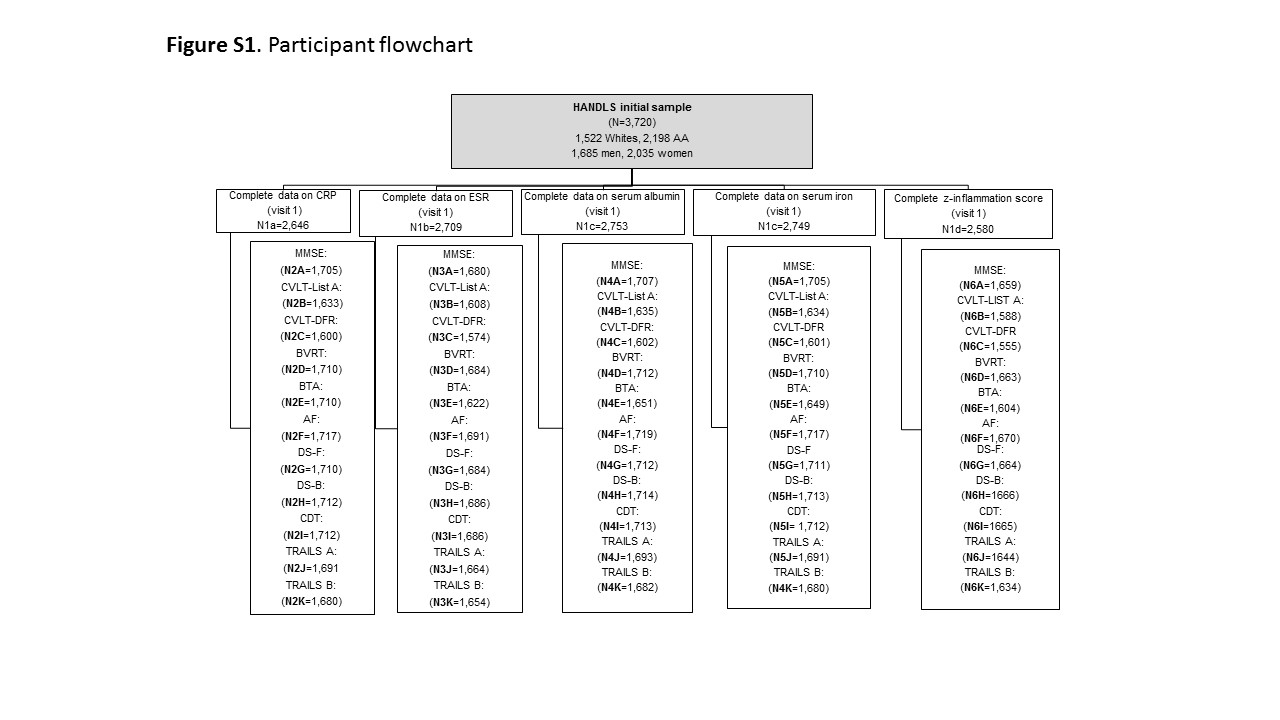

Supplement: Supplementary file 3 [file Image_1.jpg]
